# Long-term treatment with a glucagon-like peptide-1 receptor agonist reduces ethanol intake in male and female rats

**DOI:** 10.1038/s41398-020-00923-1

**Published:** 2020-07-16

**Authors:** Daniel Vallöf, Aimilia Lydia Kalafateli, Elisabet Jerlhag

**Affiliations:** Department of Pharmacology, Institute of Neuroscience and Physiology, The Sahlgrenska Academy at the University of Gothenburg, Gothenburg, Sweden

**Keywords:** Addiction, Physiology

## Abstract

Given the limited efficacy of available pharmacotherapies for treatment of alcohol use disorder (AUD), the need for new medications is substantial. Preclinical studies have shown that acute administration of glucagon-like peptide-1 receptor (GLP-1R) agonists inhibits various ethanol-related behaviours, indicating this system as a potential target for AUD. However, the effects of long-term systemic treatment of GLP-1R agonists on ethanol intake in male and female rodents are to date unknown. Therefore, we investigated the effects of 9 or 5 weeks of once weekly administration of dulaglutide, a long-acting GLP-1R agonist, on ethanol intake in male and female rats. The ethanol intake during treatment discontinuation was also monitored. In an initial attempt to identify preliminary underlying mechanisms, the effects of 9 weeks of once weekly dulaglutide treatment on monoaminergic signalling in reward-related areas were explored in both sexes. We found that 9 or 5 weeks of once weekly dulaglutide treatment reduced ethanol intake and preference in male and female rats. Following discontinuation of dulaglutide treatment, the decrease in ethanol consumption was prolonged in males, but not females. We demonstrated that 9 weeks of dulaglutide treatment differentially influenced monoaminergic signalling in reward-related areas of male and female rats. Collectively, these data imply that the GLP-1R attracts interest as a potential molecular target in the medical treatment of AUD in humans: more specifically, dulaglutide should be evaluated as a potential medication for treatment thereof.

## Introduction

Despite alcohol use disorder (AUD) being a leading cause of mortality and morbidity^1,2^, only four AUD pharmacotherapies are available. As all of these display varied efficacy^3,4^, there is a substantial need to identify new medications treating AUD. Recent translational findings suggest that these agents may include gut-brain peptides (for review see refs. ^5,6^).

The gut-brain peptide, glucagon-like peptide-1 (GLP-1) lowers blood glucose levels and inhibits glucagon secretion (for review see ref. ^7^). These insulinotropic effects contributed to the approval of GLP-1 analogues, such as the long-acting dulaglutide^8^, for treatment of diabetes type II (for review see^7^). Furthermore, GLP-1 receptor (GLP-1R) activation decreases feeding behaviours, appetite and body weight^9^. Liraglutide, another GLP-1R agonists, was therefore approved as an anti-obesity medication (for review see ref. ^10^).

This hormone has also been attributed additional physiological functions, such as attenuation of various ethanol-mediated behaviours. In male rodents, acute administration of GLP-1R agonists blocks the ability of ethanol to activate the mesolimbic dopamine system, reduces ethanol intake and prevents relapse drinking^11–15^. Additionally, repeated injections of a GLP-1R agonist reduce both consumption and operant self-administration of ethanol in male rats^15^. Despite these initial studies, it is to date unknown how long-term systemic treatment with GLP-1R agonists influences ethanol, water, and food intake and body weight in male and female rodents. Moreover, plausible mechanisms influencing these consummatory behaviours remain to be determined.

The present experiments were undertaken to investigate the ability of dulaglutide, injected once weekly for 9 or 5 weeks, to reduce ethanol intake and the preference for ethanol over water in both male and female rats. In these rats, the water and food intake as well as the body weight changes were also monitored. The protracted effect of discontinued dulaglutide treatment on these consummatory behaviours was evaluated in both sexes. The effects of 9 weeks of dulaglutide treatment on serotonin, dopamine, noradrenaline and their metabolites in brain areas known to participate in AUD processes and the behavioural responses to ethanol^16^, were explored. These findings would provide a preliminary indication of dulaglutide’s mechanisms of action for causing a reduction in ethanol-drinking behaviour. Overall, these data provide further knowledge on the GLP-1R in regards to ethanol intake in both sexes, and they constitute important findings for future clinical testing of GLP-1R agonists in AUD patients.

## Material and methods

### Animals

These experiments were approved by the Swedish Ethical Committee on Animal Research in Gothenburg (Sweden; ethical number: 1556/18). The ARRIVE (Animal Research: Reporting of In Vivo Experiments) and PREPARE (Planning Research and Experimental Procedures on Animals: Recommendations for Excellence) guidelines have been followed. Adult post-pubertal male or female outbred Rcc Han Wistar rats (approximately 150–200 g for females, and 200–240 g for males, corresponding to an approximate age of 7–8 weeks at arrival; Envigo, Horst, Netherlands) were used. Male rodents were used to allow for reproducibility and comparison between previously acquired data where male animals had been used. The inclusion of female rodents is arguably beneficial. The first week, rats were group-housed and allowed to acclimatize to the animal facilities. Thereafter, the rats were housed individually in a room with a 12-h reversed light dark cycle (lights off at 10 am) and with 20 °C and 50% humidity. The rats had free access to food and water. The number of rats included in each study was based on previous experience, where a number above 7 per treatment group is enough to show a statistically significant effect. The sample size selection was also based on previous power analysis for a significance level of 5%, an effect size of 0.2 standard deviations or more, two-tailed direction of the effect and power of study 80%. No rats were excluded in the present study, where the pre-set exclusion criteria were abnormal rodent behaviour, reduces health status or weight reduction over 15%.

### Drugs

Ethanol (95%; Solveco AB, Stockholm, Sweden) was diluted to a 20% vol/vol solution using tap water. Dulaglutide (Trulicity^®^, Kronans Apotek, Gothenburg, Sweden, a selective long-acting GLP-1R agonist (for review see ref. ^17^), was at each injection day dissolved in vehicle (0.9% NaCl). While dulaglutide was injected twice weekly in other rodent studies^8,18,19^, dulaglutide was in the present studies injected once weekly, 1 h prior to the dark cycle. The selected doses of 0.05 mg/kg or 0.1 mg/kg injected subcutaneously (sc) enhances the insulin response during graded glucose infusion^8^ and body weight^18^, and correspond to the doses used when treating patients with diabetes type II^20^. A pilot experiment was herein conducted to establish that these doses injected once weekly reduces ethanol intake (Supplementary Fig. 1). In addition, dulaglutide (0.1 mg/kg, sc) has a half-life of 1.5 days and is detected in the blood six days after an acute injection^8^. Acute dulaglutide injection has no effect per se on behaviour (Supplementary Table 1) and 9 weeks of dulaglutide treatment does not visually alter the gross motor function.

### Intermittent access 20% ethanol two-bottle-choice drinking paradigm

Rats were exposed to the intermittent access 20% ethanol two-bottle-choice drinking paradigm, as rats in this paradigm voluntarily consume high amounts of ethanol with pharmacological relevant blood ethanol concentrations^21^. In total, five separate ethanol-drinking experiments using this paradigm were carried out. For all of these, the rats had free access to one bottle of 20% ethanol and one bottle of water during three 24-h sessions per week (Mondays, Wednesdays and Fridays). During the remaining non-ethanol-drinking days, the rats had unlimited access to two water bottles. The bottles were always changed at the onset of the dark cycle. The bottles and food were weighed daily and the individual body weight was measured once weekly, allowing determination of the 24-h ethanol (g/kg), water (g), total fluid (g) and food intake (g), preference for ethanol (%, the ratio of ethanol to total fluid intake) and weekly body weight gain (the ratios of (weight each week-weight at baseline)/(weight at baseline)). In all experiments the rats consumed ethanol prior to division into treatment groups, which was done in a balanced design to establish that the baseline ethanol intake was similar in all future treatment groups. This was further done to ensure treatment groups of equal size. The drug injections and data analysis were not performed by the same individual.

#### Ethanol-drinking experiment one—effects of two different doses of dulaglutide on ethanol intake in male rats

A pilot experiment was conducted to compare two doses of dulaglutide on ethanol intake in male rats. This experiment was also conducted to establish whether once weekly injections, opposed to twice weekly in other rodent studies^8,18,19^, had an effect on ethanol consumption. Male rats consumed ethanol for 8 weeks and during this period the ethanol intake and body weight was measured. After this baseline consumption, dulaglutide (0.05 or 0.1 mg/kg (sc)) or vehicle was injected once weekly for a total of three consecutive weeks and ethanol, water, food intake and body weight were measured.

#### Ethanol-drinking experiments two and three—effects of 9 weeks of dulaglutide treatment on ethanol intake in male and female rats

As the pilot experiment revealed that a dulaglutide dose of 0.1 mg/kg reduces ethanol intake, this experiment was conducted to evaluate the ability of 9 weeks of dulaglutide treatment to reduce ethanol intake in male and female rats. Following two ethanol consumption days (baseline), male and female rats were, based on their initial ethanol intake, assigned to either dulaglutide or vehicle treatment. The male (experiment two) and the female (experiment three) rats were treated once weekly with dulaglutide (0.1 mg/kg, sc) or vehicle for nine consecutive weeks (corresponding to ethanol sessions 1–27). The rats thereafter continued in the intermittent ethanol access paradigm, but remained untreated for another 2 weeks (corresponding to ethanol sessions 28–33). Ethanol, water, food intake and body weight were measured during baseline, active treatment and during treatment discontinuation.

#### Ethanol-drinking experiments four and five—effects of 5 weeks of dulaglutide treatment on ethanol intake in male and female rats

In ethanol-drinking experiment two and three the ethanol intake was lower in rats treated with dulaglutide. In attempt to investigate the ethanol intake following treatment discontinuation in more detail, additional experiments were designed. In these ethanol-consuming rats were dulaglutide treated for 5 weeks, and the ethanol intake was monitored for an additional six untreated weeks. In detail, after two initial ethanol sessions (baseline), the male or female rats were divided into treatment groups (dulaglutide or vehicle). The male (experiment four) and the female (experiment five) rats were injected (sc) once weekly, for a total of 5 weeks, with dulaglutide (0.1 mg/kg) or vehicle (corresponding to ethanol sessions 1–15). The rats were exposed to the intermittent access ethanol paradigm for another six untreated weeks (corresponding to ethanol sessions 16–33). Ethanol, water, food intake and body weight were measured during baseline, active treatment and during treatment discontinuation.

### Ex vivo biochemical analysis

#### Biochemical experiment one and two

The aim was to gain a preliminary insight into the influence of dulaglutide on the levels of monoamines and their metabolites in reward-related areas. In this experiment, individually housed male (biochemical experiment one) and female (biochemical experiment two) rats, under ad libitum access to food and water, were treated once weekly with either dulaglutide (0.1 mg/kg, sc) or vehicle for 9 weeks. These rats were thus not ethanol consuming, in an attempt to define the effect of dulaglutide on central neurotransmission without the possible influence of variation in ethanol intake between vehicle and dulaglutide treated rats. Following euthanasia, the rats were decapitated and the brains were removed and immediately placed on a cold glass plate. The following brain areas were dissected: ventral tegmental area, prefrontal cortex, hippocampus, nucleus accumbens (NAc), striatum and amygdala. These brain areas were selected as they all participate in the behavioural response to ethanol and participate in the AUD processes^16^. Then the samples were transferred into a plastic tube and stored in −80 °C until further analysis.

#### Biochemical experiment three and four

The aim was to evaluate the effect of treatment discontinuation on neurotransmission in male and female rats consuming ethanol. For the biochemical experiment three (males from ethanol-drinking experiment four) and four (females from ethanol-drinking experiment five) the areas of ventral tegmental area, prefrontal cortex, hippocampus, NAc, striatum and amygdala were isolated as described above. All samples were placed in a plastic tube and stored in −80 °C (similarly to above experiments). These brain areas were chosen for each sex based on the results obtained from biochemical experiment one and two.

#### HPLC analysis

In brief^22^, each brain sample was homogenised (Ultrasound homogenization; Sonifier Cell Disruptor B30, Branson Sonic Power Co. Danbury, CT, USA) in a solution (0.65 mM glutathione, 5.37 mM EDTA,0.1 M perchloric acid) and subsequently centrifuged (10,000 rpm, 5 °C, 10 min). The supernatant was collected and analysed using a split fraction HPLC-ED system. Dopamine, noradrenaline, and serotonin were analysed with an ion-exchange column (Nucleosil; 5 μ SA 100 A, 150 × 2 mm; Phenomenex, Torrance, CA, USA) and a mobile phase (40 mg EDTA, 5.84 g NaOH, 13.3 g citric acid, 200 ml methanol, and distilled water to a total volume of 1000 ml). DOPAC, 5-HIAA and 3-MT were measured with a reverse phase column (Nucleosil, 3 μ, C18, 100 A, 50 × 2 mm; Phenomenex) and a mobile phase (40 mg EDTA, 3.02 g dipotassium phosphate, 11.22 g citric acid, 60 ml methanol, and distilled water to a total volume of 1000 ml). Electrochemical detection (amperometric detectors; Waters 460) was applied and the currents were recorded (Dionex Chromeleon software package; Dionex, Sunnyvale, CA, USA).

### Statistical analysis

Baseline values (before treatment initiation) were evaluated with one-way ANOVA (ethanol-drinking experiment one) or two-tailed unpaired t-test (ethanol-drinking experiment two-five). Data from all five intermittent access ethanol-drinking experiment were analysed with repeated two-way ANOVA followed by Bonferroni post-hoc test for comparisons between treatments and given time points (adjusted for multiple testing; the analysis was divided into active treatment and treatment discontinuation for ethanol-drinking experiment 2–5). The ability of dulaglutide to reduce ethanol intake between sexes ((mean ethanol intake for dulaglutide for each session—mean ethanol intake for vehicle for each session)_MALES_ compared to (mean ethanol intake for dulaglutide for each session—mean ethanol intake for vehicle for each session)_FEMALES_) and the biochemical data were evaluated with two-tailed unpaired t-test. Data were always presented as mean ± SEM. A comprehensive statistical analysis is presented in Supplementary Figs. 1–11 and Supplementary Tables 1–8. Analysis of the data, all *n* ≥ 5, was blinded to the statistician. In total each experiment was conducted once. However, given that ethanol-drinking experiment 1, 2 and 4 as well as ethanol-drinking experiment 3 and 5 share doses, drugs and some timing, they might be considered to serve as their own replications. All the data sets were normally distributed.

## Results

### Effects of two dulaglutide doses on ethanol intake in male rats (ethanol-drinking experiment one)

During baseline ethanol-drinking there were no differences in ethanol intake (F(2,42)=0.07, *P* = 0.9327, *n* = 15 per group) in male rats later subjected to vehicle, or either dose of dulaglutide (0.05 or 0.1 mg/kg).

The statistical analysis for all parameters is presented in Supplementary Fig. 1. In male rats, both doses of dulaglutide decreased ethanol intake (Supplementary Fig. 1A). Dulaglutide treatment did not affect ethanol preference (Supplementary Fig. 1B), water intake (Supplementary Fig. 1C) or total fluid intake (Supplementary Fig. 1D). Dulaglutide treatment decreased food intake (Supplementary Fig. 1E) and prevented the body weight gain (Supplementary Fig. 1F). As further evident in Supplementary Fig. 1E, F, the effects on food intake and body weight gain are greater in rats treated with 0.1 mg/kg than with 0.05 mg/kg. Neither dose of dulaglutide induce any visual alterations in gross behaviour. Therefore, the higher dose (0.1 mg/kg) was used in future studies.

### Effects of 9 weeks of dulaglutide treatment on ethanol intake in male rats (ethanol-drinking experiment two)

**Fig. 1:**
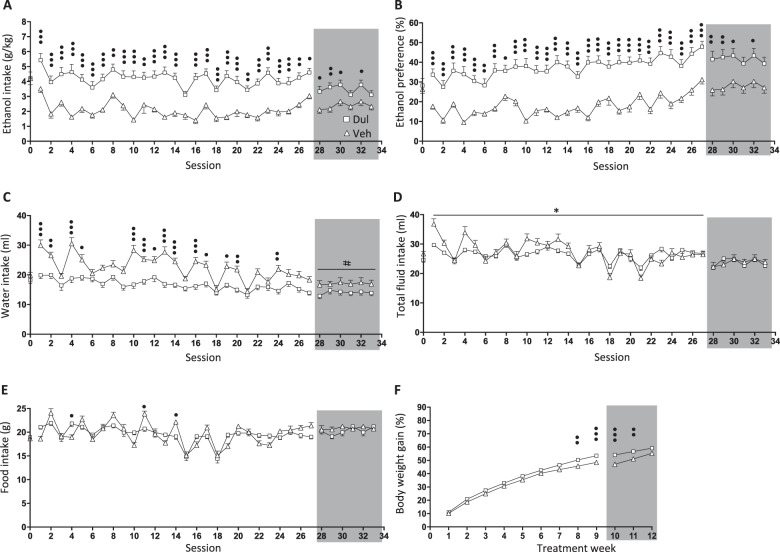
Dulaglutide treatment for 9 weeks decreased ethanol intake in male rats. **a** Compared to vehicle (Veh, square), dulaglutide (Dul, triangle) treatment for 9 weeks (sessions 1–27) reduced ethanol intake in male rats. After treatment discontinuation (sessions 28–33, area marked with grey), alcohol intake was lower in the previously dulaglutide-treated group compared to vehicle. **b** In comparison to vehicle, dulaglutide decreased ethanol preference during treatment as well as after termination. **c** Water intake in dulaglutide-treated rats was higher compared to vehicle, an effect that was sustained after discontinuation of treatment. **d** Compared to vehicle, repeated dulaglutide treatment increased total fluid intake only during active treatment period. **e** Active dulaglutide altered food intake, however it was similar in the groups previously treated with dulaglutide or vehicle. **f** Dulaglutide reduced body weight gain, an effect which persisted after treatment discontinuation. Data are presented as mean ± SEM; *P < 0.05: interaction effects after repeated measures two-way ANOVA, ^#^*P* < 0.05: treatment effects after repeated measures two-way ANOVA, ^•^*P* < 0.05, ^••^*P* < 0.001, ^•••^*P* < 0.001: Bonferroni post-hoc analysis. Session 0 represent the baseline values.

There were no differences in baseline ethanol intake (*P* = 0.9941, *n* = 25 per group), ethanol preference (*P* = 0.6374), water intake (*P* = 0.2610), total fluid intake (*P* = 0.0991), food intake (*P* = 0.6007) or body weight (*P* = 0.2537), in male rats later subjected to vehicle or dulaglutide (0.1 mg/kg) treatment for 9 weeks (Fig. 1).

A comprehensive statistical analysis for each parameter, during active treatment and at treatment discontinuation, is described in Supplementary Table 2. Nine weeks of dulaglutide reduced ethanol intake (Fig. 1a) and ethanol preference (Fig. 1b), effects which persisted following discontinuation of treatment. Possibly as compensation, dulaglutide treatment increased water intake (Fig. 1c), accompanied by increased total fluid intake (Fig. 1d). This increase in water intake was also evident following termination of treatment. Dulaglutide both increased and decreased food intake at different sessions (Fig. 1e). The body weight gain was lower in dulaglutide treated rats, and remained lower following discontinuation of treatment in rats previously treated with dulaglutide (Fig. 1f).

### Effects of 9 weeks of dulaglutide treatment on ethanol intake in female rats (ethanol-drinking experiment three)

**Fig. 2:**
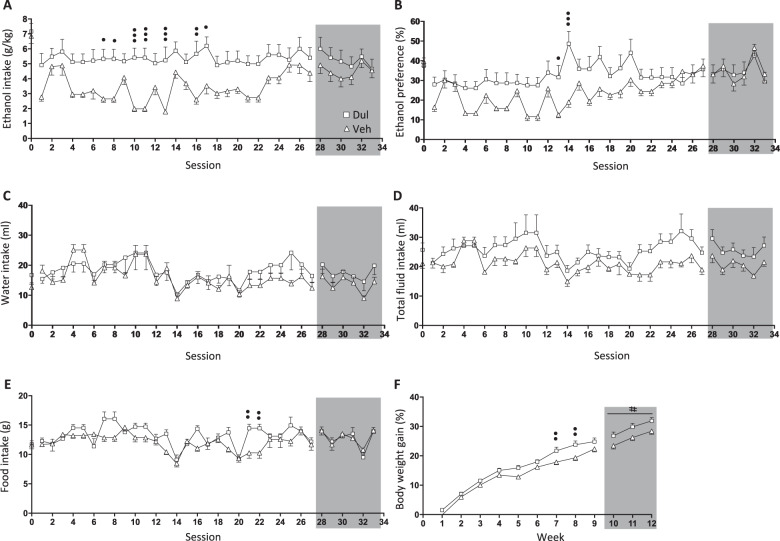
Nine weeks of dulaglutide treatment reduced ethanol intake in female rats. **a** Compared to vehicle (Veh, square), repeated dulaglutide treatment (Dul, triangle) decreased ethanol intake in female rats during active treatment (session 1–27). After treatment discontinuation (session 28–33, area marked with grey) ethanol intake did not differ between the dulaglutide and vehicle groups. **b** Dulaglutide treatment reduced ethanol preference, however this effect did not persist after treatment termination. Dulaglutide did not affect (**c**) water or (**d**) total fluid intake. **e** Dulaglutide decreased food intake, but this difference was not evident after treatment termination. **f** Dulaglutide treatment reduced the body weight gain. This overall lower body weight gain sustained post-treatment. ^#^*P* < 0.05: treatment effects after repeated measures two-way ANOVA, ^•^*P* < 0.05, ^••^*P* < 0.001, ^•••^*P* < 0.001: Bonferroni post-hoc analysis. Session 0 represent the baseline values.

There were no differences in baseline ethanol intake (*P* = 0.6720, *n* = 10 per group), ethanol preference (*P* = 0.6397), water intake (*P* = 0.1593), total fluid intake (*P* = 0.1286), food intake (*P* = 0.9023) or body weight (*P* = 0.7309), in female rats later subjected to vehicle or dulaglutide (0.1 mg/kg) treatment for 9 weeks (Fig. 2).

Supplementary Table 3 shows a comprehensive statistical analysis for each parameter during active treatment and at treatment discontinuation. Nine weeks of dulaglutide reduced ethanol intake (Fig. 2a) and ethanol preference (Fig. 2b), however the effects did not persist following discontinuation of treatment. Neither the water intake (Fig. 2c) or the total fluid intake (Fig. 2d) were influenced by dulaglutide treatment in female rats. Dulaglutide reduced food intake, however this difference was not evident after treatment was terminated (Fig. 2e). Moreover, dulaglutide prevented the body weight gain in female rats, an effect that persisted after treatment was discontinued (Fig. 2f).

### Effects of 5 weeks of dulaglutide treatment on ethanol intake in male rats (ethanol-drinking experiment four)

**Fig. 3:**
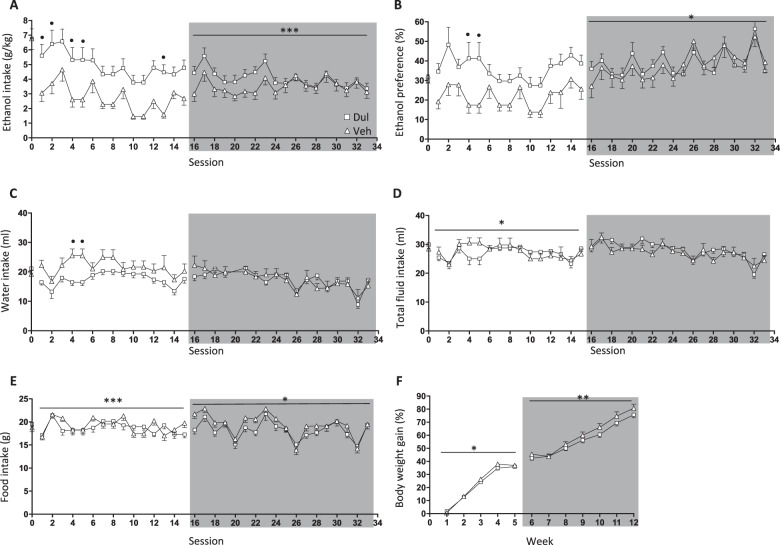
Dulaglutide exposure for 5 weeks decreased ethanol consumption in male rats. **a** Compared to vehicle (Veh, square), repeated dulaglutide treatment (Dul, triangle) decreased ethanol intake in male rats during the active treatment period (session 1–15). After treatment discontinuation (session 16–33, area marked with grey) ethanol intake was initially lower in the previously dulaglutide-treated group. **b** Dulaglutide decreased ethanol preference, and this difference was also initially evident after treatment discontinuation. Dulaglutide increased (**c**) water intake and (**d**) total fluid intake. However, these differences did not persist during the treatment discontinuation period. **e** There was an overall difference in food intake between the two groups of rats, an effect evident both during treatment and at discontinuation. **f** Dulaglutide overall decreased the body weight gain, an overall effect which persisted after treatment termination. Data are presented as mean ± SEM; *P < 0.05, **P < 0.01, ***P < 0.001: interaction effects after repeated measures two-way ANOVA. ^•^*P* < 0.05: Bonferroni post-hoc analysis. Session 0 represent the baseline values.

There were no differences in baseline ethanol intake (*P* = 0.9999, *n* = 10 per group), ethanol preference (*P* = 0.6120), water intake (*P* = 0.3441), total fluid intake (*P* = 0.3452), food intake (*P* = 0.4341) or body weight (*P* = 0.1186), in male rats later subjected to vehicle or dulaglutide (0.1 mg/kg) treatment for 5 weeks (Fig. 3).

Supplementary Table 4 displays a comprehensive statistical analysis for each parameter during active treatment and at treatment discontinuation. In male rats, 5 weeks of dulaglutide treatment reduced ethanol intake, an effect evident during treatment discontinuation (Fig. 3a). The ethanol preference was lower in male rats during active dulaglutide treatment and after treatment termination (Fig. 3b). Dulaglutide treatment increased water intake (Fig. 3c), accompanied by increased total fluid intake (Fig. 3d). These effects did not persist after treatment discontinuation. Dulaglutide also increased food intake, an increase which persisted during treatment discontinuation (Fig. 3e). Dulaglutide treatment elevated the body weight gain, and this effect was evident after treatment termination (Fig. 3f).

### Effects of 5 weeks of dulaglutide treatment on ethanol intake in female rats (ethanol-drinking experiment five)

**Fig. 4:**
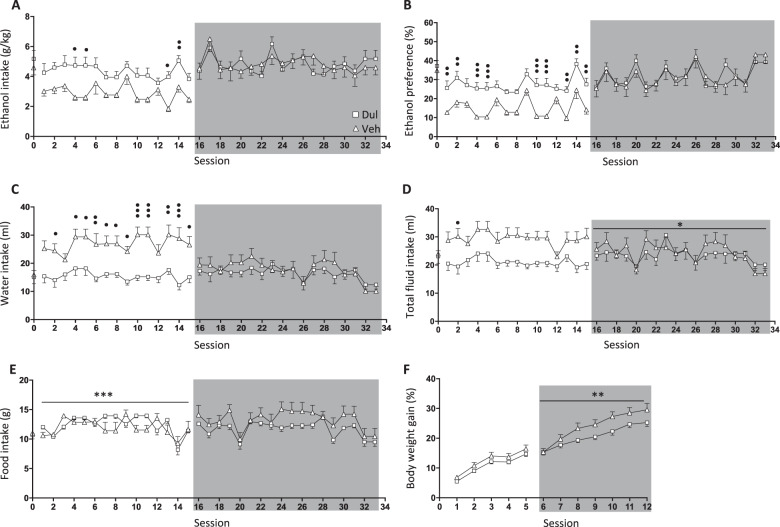
Five weeks of dulaglutide treatment decreased ethanol consumption in female rats. **a** Compared to vehicle (Veh, square), dulaglutide (Dul, triangle) reduced ethanol intake in female rats during 5 weeks of active treatment (sessions 1–15). Ethanol consumption returned to baseline after treatment was terminated (session 16–33, area marked with grey). **b** Dulaglutide decreased ethanol preference, but no differences were noted during the treatment discontinuation period. **c** Water intake was higher in dulaglutide-treated rats, but this difference did not persist after discontinuation of treatment. **d** Dulaglutide treatment increased total fluid intake, which was sustained during the treatment discontinuation period. **e** Dulaglutide had an overall effect on food intake. However, this difference was not evident following treatment discontinuation. **f** Active dulaglutide treatment had no effect on body weight gain. Body weight gain was overall higher in rats previously treated with dulaglutide. Data are presented as mean ± SEM; *P < 0.05, **P < 0.01, ***P < 0.001: interaction effects after repeated measures two-way ANOVA. ^•^*P* < 0.05, ^••^*P* < 0.001, ^•••^*P* < 0.001: Bonferroni post-hoc analysis. Session 0 represent the baseline values.

There were no differences in baseline ethanol intake (*P* = 0.4395, *n* = 10 per group), ethanol preference (*P* = 0.6606), water intake (*P* = 0.7542), total fluid intake (*P* = 0.7565), food intake (*P* = 0.6132) or body weight (*P* = 0.5784), in female rats later subjected to vehicle or dulaglutide (0.1 mg/kg) treatment for 5 weeks (Fig. 4).

A comprehensive statistical analysis for each parameter, during active treatment and at treatment discontinuation, is described in Supplementary Table 5. Dulaglutide reduced ethanol intake in female rats and it returned to baseline after treatment termination (Fig. 4a). Dulaglutide treatment decreased ethanol preference (Fig. 4b), but only during active treatment. There was an increase in water intake in dulaglutide treated rats, but not after treatment termination (Fig. 4c). Total fluid intake was increased in rats treated with dulaglutide and persisted following treatment discontinuation (Fig. 4d). Active dulaglutide treatment influenced food intake, by either a decrease or increase, but there were no differences between the previously treated rats (Fig. 4e). Active treatment did not influence the body weight gain, but the body weight gain was elevated in the rats previously treated with dulaglutide (Fig. 4f).

### Differences in the magnitude in ability of dulaglutide to reduce ethanol intake between male and female rats

Dulaglutide treatment reduced ethanol intake in both sexes. Supplementary Table 6 shows the average difference in ethanol intake over time between vehicle and dulaglutide (9 or 5 weeks) treatment in male and female rats, and further that dulaglutide reduced ethanol intake more profoundly in male than female rats at both treatment intervals.

### Effects of 9 weeks of dulaglutide treatment on the ex vivo concentrations of monoamines and their metabolites of male rats (biochemical experiment one) and female rats (biochemical experiment two)

**Fig. 5:**
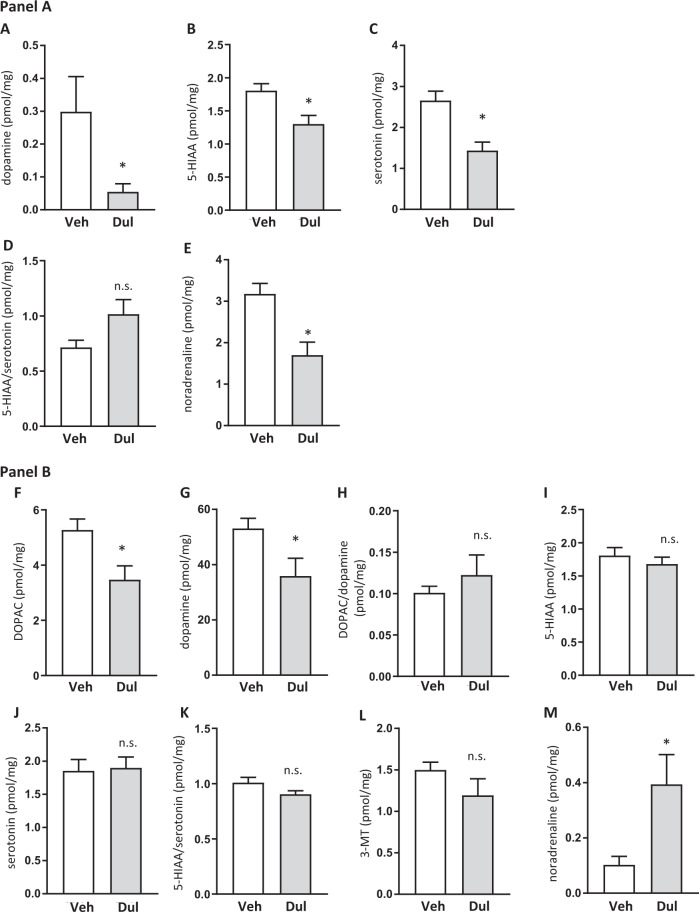
Dulaglutide treatment for 9 weeks alters the levels of monoamines and their metabolites in the amygdala of male and striatum of female rats. **a** shows the levels of monoamines and their metabolites in the amygdala of male rats treated with dulaglutide (Dul) or vehicle (Veh). (**a**) Dopamine, **b** 5-HIAA and **c** serotonin levels were decreased in Dul-treated rats. **d** Dulaglutide had no effect on the 5-HIAA/serotonin turnover, but (**e**) reduced noradrenaline. **b** shows the levels of monoamines and their metabolites in the striatum of female rats treated with Dul or Veh. Dul reduced (**f**) DOPAC and **g** dopamine, but did not affect (**h**) DOPAC/dopamine turnover, **i** 5-HIAA, **j** serotonin (**k**) 5-HIAA/serotonin turnover or **l** 3-MT. **m** Dulaglutide treatment increased noradrenaline levels. Data are presented as mean ± SEM; *P < 0.05, n.s.: not significant after unpaired *t*-test.

Compared to vehicle (*n* = 10), dulaglutide (*n* = 10) treatment decreased dopamine (*P* = 0.0319; Fig. 5a), 5-HIAA (*P* = 0.0077; Fig. 5b) and serotonin (*P* = 0.0010; Fig. 5c), but did not alter 5-HIAA/serotonin turnover (*P* = 0.0644; Fig. 5d) in the amygdala of male rats. In the same area, dulaglutide decreased noradrenaline (*P* = 0.0022; Fig. 5e). The comprehensive statistical analysis for the remaining brain areas is presented in Supplementary Fig. 2–6. Dulaglutide did not alter monoamine levels or their metabolites in striatum (Supplementary Fig. 2), hippocampus (Supplementary Fig. 3) or ventral tegmental area (Supplementary Fig. 4). Dulaglutide decreased DOPAC and dopamine in the NAc (Supplementary Fig. 5), and reduced DOPAC and dopamine turnover in the prefrontal cortex (Supplementary Fig. 6).

Analysis of the striatum of female rats showed that dulaglutide (*n* = 10), compared to vehicle (*n* = 10) treatment reduced DOPAC (*P* = 0.0115; Fig. 5f) and dopamine (*P* = 0.0321; Fig. 5g). However, dulaglutide did not change DOPAC/dopamine turnover (*P* = 0.4127; Fig. 5h), 5-HIAA (*P* = 0.4161; Fig. 5i), serotonin (*P* = 0.8596; Fig. 5j), 5-HIAA/serotonin turnover (*P* = 0.07759; Fig. 5k) or 3-MT (*P* = 0.1821; Fig. 5l). Dulaglutide treatment increased noradrenaline (*P* = 0.0179; Fig. 5m). The comprehensive statistical analysis for the remaining brain areas is presented in Supplementary Figs. 7–11. Dulaglutide did not change the monoamine levels or their metabolites in amygdala (Supplementary Fig. 7), hippocampus (Supplementary Fig. 8), NAc (Supplementary Fig. 9) or ventral tegmental area (Supplementary Fig. 10), but reduced DOPAC in the prefrontal cortex (Supplementary Fig. 11).

### Effects of discontinuation of dulaglutide treatment on the ex vivo levels of monoamines and their metabolites in male (biochemical experiment three) and female rats (biochemical experiment four)

As shown in Fig. 3a (male rats) and 4a (female rats), the ethanol consumption at ethanol-drinking session 33 was similar in rats previously treated with dulaglutide or vehicle for 5 weeks. In these ethanol-drinking rats which at session 33 were untreated for six weeks, there were no differences in the monoamines and their metabolites levels in the studied brain areas (Data and comprehensive statistical analysis are found in Supplementary Table 7 for males or Supplementary Table 8 for females, *n* = 10 for all groups) in rats previously treated with dulaglutide or vehicle.

## Discussion

In both male and female rats 9 or 5 weeks of once weekly injections with dulaglutide decreased ethanol intake, which was accompanied by a reduction in ethanol preference. Male rats dulaglutide injected for 5 or 9 weeks, and female rats treated for 5 weeks compensated for their reduction in ethanol intake by enhancing their water intake. Albeit these are the first ethanol intake experiments in male and female rats exposed to a GLP-1R agonist for longer periods of time, they are consistent with previous studies demonstrating that acute or sub-chronic administration of different GLP-1R agonists reduce ethanol consummatory behaviours. Specifically, in male rodents GLP-1R agonists decrease ethanol intake, prevent relapse drinking, decline the number of drinking bouts and decrease both oral and intravenous operant self-administration ethanol^11–15,23^. Similarly, in non-human male primates, sub-chronic GLP-1R agonist treatment reduces ethanol intake^24^. Clinical relevance to these data might be added by a human genetic study that found association between polymorphisms in the GLP-1R gene and AUD prevalence as well as behavioural responses to ethanol^13^. Here we further found that male rats previously treated with dulaglutide display a lower ethanol intake and ethanol preference for an additional untreated 3 weeks. These findings are consistent with previous studies reviling that male rodents that previously received either of two other GLP-1R agonists, liraglutide or AC3174, continued to self-administer or consume less ethanol^13,15^. Studies exploring the effect of long-term treatment with other clinically used anti-diabetic GLP-1R drugs on ethanol intake in rodents are thus warranted.

In this study, we observed reduced ex vivo dopaminergic neurotransmission in the amygdala of male and in the striatum of female rats treated with dulaglutide once weekly for 9 weeks. Although the nature of these data is preliminary, the decrease in dopamine signalling by dulaglutide could possibly prevent the ability of ethanol to increase dopamine levels in the aforementioned areas^25–32^. Supportively, male mice acutely treated with the GLP-1R agonists exendin-4 or liraglutide, do not display dopamine release in the NAc following an acute ethanol injection^11,15^. However, the ability of dulaglutide to reduce ethanol intake may involve additional neurotransmission as our ex vivo data also show reduced serotonergic and noradrenergic neurotransmission in the amygdala of males and noradrenaline in the striatum of females. It should however be noted that these rats did not consume ethanol in attempt to define the effects of dulaglutide on central neurotransmission without the possible interaction with ethanol. All together this should be considered as a limitation and such studies are warranted for the future. We further revealed that these alterations in central neurotransmission during active dulaglutide treatment, just as ethanol-drinking behaviour, disappears after 6 weeks of treatment discontinuation. As the present findings preliminary pin point areas and neurotransmitters possibly contributing to the ability of dulaglutide to reduce ethanol intake, the downstream mechanisms of dulaglutide should be evaluated in more detail in upcoming studies. Future studies should also elucidate the effects of dulaglutide on other brain areas and neurotransmitters that participate in AUD processes^33^.

Even though we here suggest that dulaglutide decreases ethanol intake by alterations of neurotransmission in reward-related areas other factors might influence the obtained data. One of these could be malaise, a common side effect by GLP-1R agonists, including dulaglutide^34^. However, this appears less likely as clinical studies show that dulaglutide induces less nausea than other GLP-1R agonists^35^, and that we here report that dulaglutide does not reduce the water intake. Besides, the baseline values on ethanol and water intake are different between the different ethanol-drinking experiments and between sexes. This inconsistency in baseline behavioural might be a confounding factor, which is diminished by including vehicle controls in each independent series of experiments. Moreover, the approximate age at initiation of the experiment was 9–10 weeks, and the possibility that older ethanol-consuming rats respond differently to dulaglutide should be considered. In the present studies rats were injected sc once weekly, and as sc injections might affect behaviour and central neurotransmission the omission of non-injected rats should be considered as a limitation. Altered locomotor activity could tentatively influence the ability of dulaglutide to reduce ethanol intake. Albeit, this appears less likely as we here show that an acute dulaglutide injection did not affect locomotor activity and that 9 weeks of dulaglutide treatment did not visually alter the gross motor function. However, the lack of locomotor activity measurements in the long-term treated rats should be considered as a limitation. Even though rats exposed to the intermittent access model voluntarily consume high amounts of ethanol that reaches pharmacological relevant blood ethanol concentrations^21^, rats herein do not escalate their ethanol intake over time. As this is one out of 11 AUD criteria, the possibility should be considered that this model does not reflect AUD. While other rodent studies inject dulaglutide twice weekly^8,18,19^, dulaglutide was herein injected once weekly as this correspond to the clinical use^20^. Raising the possibility that low and inefficient plasma levels of dulaglutide are reach at the end of each week, and that steady state concentrations are not reached. The lack of data identifying the concentrations of dulaglutide throughout this experiment should thus be considered a limitation. It should however be considered that one injection of dulaglutide reduces the blood glucose levels, an effect also evident at steady state concentrations reached after 2–4 weeks of injections^20^ and that dulaglutide is detected in the blood six days after an acute sc injection^8^. It is also possible that lower doses of dulaglutide more efficiently reduces ethanol intake than those known to alter the blood glucose levels as it has been shown for liraglutide^15,36^. In support for this contention are the present data demonstrating that dulaglutide equally reduces ethanol consumption on each ethanol consummatory day, independent on the time from injection.

Albeit dulaglutide reduced ethanol drinking in both sexes, we found that once weekly injection with dulaglutide differently affected ethanol intake between male and female rats. Firstly, dulaglutide reduced ethanol intake more profoundly in males compared to female rats. Secondly, whereas there was a reduction in ethanol intake at the entire post-9-week dulaglutide period in male rats, the ethanol-drinking behaviour returns to baseline after a week in female rats. Thirdly, the ethanol consumption returned to baseline immediately after treatment discontinuation in 5 weeks of dulaglutide-treated females, while it remained decreased for an additional 3 weeks in males. Fourthly, there appear to be a tolerance development towards the end of the treatment in female, but not male, rats treated with dulaglutide for 9 weeks. A possible explanation for these sex differences could be the observed sex-specific differences in central neurotransmission following once weekly dulaglutide treatment. Other possibilities could be that these parameters are affected inversely by the pharmacokinetic properties of dulaglutide which could differ between male and female rats or that females are less sensitive to GLP-1R agonists. Supportively, exendin-4 into the lateral hypothalamus causes less pronounced reduction in food intake and inhibits operant conditioning for sucrose in female, compared to male rats^37^. Different hormonal status between sexes is another factor that might influence the response to dulaglutide.

Neither males nor females displayed tolerance to 5 weeks of dulaglutide treatment which is in line with the data displaying that eight days of treatment with exendin-4, attenuates relapse drinking in male mice without inducing a tolerance effect^14^. On the other hand, eight days of treatment with liraglutide induced a tolerance effect on ethanol intake in male rats^15^ and in male non-human primates^24^. In contrast to male rats treated with dulaglutide for 9 weeks, female rats appear to develop tolerance toward the end of the treatment paradigm. Although these differences in tolerance development between sexes and the GLP-1R agonists is unknown, a possible explanation may lie in their ability to penetrate and activate different brain areas^38–40^. Immunogenicity towards dulaglutide is reported to be low^41^, however a sex-dependent difference in development of anti-drug antibodies towards dulaglutide might influence the observed sex differences.

Towards the end of 9 weeks of treatment, once weekly dulaglutide treatment prevented the body weight gain observed in vehicle treated male and female rats. This effect persisted after treatment discontinuation. This reduction of body weight gain by dulaglutide is in line with previous reports demonstrating that exendin-4 or liraglutide reduced body weight^15,42–44^. We further observed that the body weight gain was enhanced in rats previously treated with dulaglutide for 5 weeks. The rational for these differences in body weight response following dulaglutide treatment remains to be evaluated, but falls outside the scope of the present study. Previous studies in male rodents report decrease in chow intake following pharmacological activation of GLP-1R^15,45–47^. Nevertheless, no consistent effect of dulaglutide on chow intake in either sex was noted in this study. It is therefore possible that dulaglutide decreases the most rewarding substrate when exposed to a choice of chow and ethanol, as previously shown with other gut-brain peptides such as ghrelin. Accordingly, ghrelin receptor antagonists do not reduce chow intake in a chow-ethanol-peanut butter choice situation^48,49^.

Collectively, long-term once weekly dulaglutide treatment decreased ethanol intake and ethanol preference in both male and female rats. This decline was also evident during the post-treatment periods in males, but not females. Moreover, dulaglutide altered the levels of monoamines and their metabolites in reward-related brain areas, including amygdala in males and striatum in females. Although the present study does not model diabetes, ethanol intake should be monitored in dulaglutide treated diabetic type II patients and in obese individuals treated with liraglutide^50^. Indeed, a small and preliminary study report that liraglutide reduces ethanol intake in patients with type II diabetes. This would provide a preliminary clinical indication towards dulaglutide’s ability to influence ethanol intake in patients with AUD, which then should be investigated properly in clinical trials. Indeed, as integrating pharmacological and behavioural treatments are essential when treating patients with AUD^51^, once weekly treatment with dulaglutide, with our without behavioural treatment, attracts interest as a potential target for the treatment of AUD.

## Supplementary information

Supplementary Tables

Supplementary Figures
