# Supplementary material for: Long-term treatment with a glucagon-like peptide-1 receptor agonist reduces ethanol intake in male and female rats
Source: Transl Psychiatry. 2020 Jul 16;10:238. doi: 10.1038/s41398-020-00923-1 (PMC7367312; doi:10.1038/s41398-020-00923-1)
Supplement: Supplementary file 1 — Supplementary Tables [file 41398_2020_923_MOESM1_ESM.docx]

**Supplementary Table 1. Effects of an acute dulaglutide treatment on locomotor activity in male and female rats**

|  |  | **Males** | **Females** |
| --- | --- | --- | --- |
| **Ambulatory distance**  **habituation (cm)** | veh | 2179±256 | 5341±466 |
|  | dul | 2143±173 | 4902±776 |
|  | *p-value* | 0.9025 | 0.6343 |
| **Ambulatory distance**  **after treatment (cm)** | veh | 420±139 | 605±199 |
|  | dul | 432±210 | 649±290 |
|  | *p-value* | 0.2471 | 0.9085 |

The activity of male or female rats was recorded in sound-attenuated, that were placed in ventilated and dim lit locomotor boxes (420 x 420 x 200 mm; Open Field Activity System; Med Associates Inc; Georgia, Vermont, USA). 15 x 15 infrared beams at the bottom of the floor detected the rat’s movement and a computer program recorded the rat’s distances travelled (cm per 5 minutes) during the experiment. The rats (n=9 per treatment group) habituated to the boxes for 60 minutes and were then injected with either dulaglutide (dul; 0.1 mg/kg, sc) or a similar volume of vehicle (veh). The subsequent 60-minute cumulative locomotor activity was thereafter registered in an attempt to evaluate the effect of dulaglutide treatment on locomotor activity *per se*. The male and female rats were exposed to identical protocols, but were tested on different experimental days.

**Supplementary Table 2. Effects of nine weeks of dulaglutide treatment on consummatory behaviour in male rats**

|  |  | **Repeated measures two-way ANOVA** | **Bonferroni post-hoc test** |
| --- | --- | --- | --- |
| **Active treatment period** | **Alcohol intake (g/kg)**  **Figure 1A** | Treatment: F(1,48)=41.75, P<0.0001 | P<0.05, s27  P<0.01, s8, s11, s21, s26  P<0.001, s1-7, s9-10, s12-14, s16-20, s22-25 |
|  |  | Time: F(26,1248)=9.31, P<0.0001 |  |
|  |  | Interaction: F(26,1248)=2.21, P<0.0001 |  |
|  | **Alcohol preference (%)**  **Figure 1B** | Treatment: F(1,48)=43.76, P<0.0001 | P<0.05, s8  P<0.01, s6  P<0.001, s1-5, s7, s9-27 |
|  |  | Time: F(26,1248)=14.03, P<0.0001 |  |
|  |  | Interaction: F(26,1248)=3.22, P<0.0001 |  |
|  | **Water intake (ml)**  **Figure 1C** | Treatment: F(1,48)=19.58, P<0.0001 | P<0.05, s5, s12, s17, s19  P<0.01, s2, s20, s24  P<0.001, s1, s4, 10-11, s13-14, s16 |
|  |  | Time: F(26,1248)=20.70, P<0.0001 |  |
|  |  | Interaction: F(26,1248)=6.00, P<0.0001 |  |
|  | **Total intake (ml)**  **Figure 1D** | Treatment: F(1,48)=0.48, P=0.4912 | n.s., s1-27 |
|  |  | Time: F(26,1248)=21.95, P<0.0001 |  |
|  |  | Interaction: F(26,1248)=5.45, P<0.0001 |  |
|  | **Food intake (g)**  **Figure 1E** | Treatment: F(1,48)=0.15, P=0.6957 | P<0.05, s4, s11, s14 |
|  |  | Time: F(26,1248)=20.45, P<0.0001 |  |
|  |  | Interaction: F(26,1248)=4.91, P<0.0001 |  |
|  | **Body weight gain (%)**  **Figure 1F** | Treatment: F(1,48)=7.24, P=0.0098 | P<0.01, s8  P<0.001, s9 |
|  |  | Time: F(8,384)=1372.00, P<0.0001 |  |
|  |  | Interaction: F(8,384)=3.10, P=0.0021 |  |
| **Discontinued treatment period** | **Alcohol intake (g/kg)**  **Figure 1A** | Treatment: F(1,48)=9.61, P=0.0032 | P<0.05, s28, s30, s32  P<0.01, s29 |
|  |  | Time: F(5,240)=7.03, P<0.0001 |  |
|  |  | Interaction: F(5,240)=1.88, P=0.09 |  |
|  | **Alcohol preference (%)**  **Figure 1B** | Treatment: F(1,48)=10.63, P=0.0021 | P<0.05, s30, s32  P<0.01, s28-29 |
|  |  | Time: F(5,240)=2.34, P=0.0422 |  |
|  |  | Interaction: F(5,240)=0.64, P=0.6766 |  |
|  | **Water intake (ml)**  **Figure 1C** | Treatment: F(1,48)=4.20, P=0.0459 | n.s., s28-33 |
|  |  | Time: F(5,240)=0.69, P=0.6320 |  |
|  |  | Interaction: F(5,240)=0.27, P=0.9331 |  |
|  | **Total intake (ml)**  **Figure 1D** | Treatment: F(1,48)=0.00, P=0.9693 |  |
|  |  | Time: F(5,240)=4.25, P=0.0010 |  |
|  |  | Interaction: F(5,240)=1.31, P=0.2588 |  |
|  | **Food intake (g)**  **Figure 1E** | Treatment: F(1,48)=1.45, P=0.2344 |  |
|  |  | Time: F(5,240)=1.31, P=0.2593 |  |
|  |  | Interaction: F(5,240)=1.55, P=0.1759 |  |
|  | **Body weight gain (%)**  **Figure 1F** | Treatment: F(1,48)=10.07, P=0.0026 | P<0.01, s11  P<0.001, s10 |
|  |  | Time: F(2,96)=422.00, P<0.0001 |  |
|  |  | Interaction: F(2,96)=22.39, P<0.0001 |  |

Statistical analysis of the effects of nine weeks of dulaglutide treatment (drinking session 1-27) and treatment discontinuation (drinking session 28-33) on all measured parameters in male rats; s: drinking session.

**Supplementary Table 3. Effects of nine weeks of dulaglutide treatment on consummatory behaviour in female rats**

|  |  | **Two-way ANOVA, repeated measures** | **Bonferroni post-hoc test** |
| --- | --- | --- | --- |
| **Active treatment period** | **Alcohol intake (g/kg)**  **Figure 2A** | Treatment: F(1,18)=11.51, P=0.0032 | P<0.05, s7, s8, S17  P<0.01, s16  P<0.001, s10-11, s13 |
|  |  | Time: F(26,468)=4.46, P<0.0001 |  |
|  |  | Interaction: F(26,468)=2.51, P<0.0001 |  |
|  | **Alcohol preference (%)**  **Figure 2B** | Treatment: F(1,18)=5.34, P=0.0328 | P<0.05, s13  P<0.001, S14 |
|  |  | Time: F(26,468)=6.62, P<0.0001 |  |
|  |  | Interaction: F(26,468)=3.18, P<0.0001 |  |
|  | **Water intake (ml)**  **Figure 2C** | Treatment: F(1,18)=0.75, P=0.3973 |  |
|  |  | Time: F(26,468)=5.36, P<0.0001 |  |
|  |  | Interaction: F(26,468)=1.11, P=0.3258 |  |
|  | **Total intake (ml)**  **Figure 2D** | Treatment: F(1,18)=4.03, P=0.0599 |  |
|  |  | Time: F(26,468)=4.04, P<0.0001 |  |
|  |  | Interaction: F(26,468)=0.88, P=0.6415 |  |
|  | **Food intake (g)**  **Figure 2E** | Treatment: F(1,18)=4.57, P=0.0464 | P<0.01, s21-22 |
|  |  | Time: F(26,468)=7.73, P<0.0001 |  |
|  |  | Interaction: F(26,468)=2.88, P<0.0001 |  |
|  | **Body weight gain (%)**  **Figure 2F** | Treatment: F(1,18)=6.72, P=0.0184 | P<0.01, s7-8 |
|  |  | Time: F(8,144)=423.50, P<0.0001 |  |
|  |  | Interaction: F(8,144)=2.70, P=0.0085 |  |
| **Discontinued treatment period** | **Alcohol intake (g/kg)**  **Figure 2A** | Treatment: F(1,18)=0.88, P=0.3609 |  |
|  |  | Time: F(5,90)=4.25, P=0.0016 |  |
|  |  | Interaction: F(5,90)=1.22, P=0.3076 |  |
|  | **Alcohol preference (%)**  **Figure 2B** | Treatment: F(1,18)=0.00, P=0.9368 |  |
|  |  | Time: F(5,90)=8.76, P<0.0001 |  |
|  |  | Interaction: F(5,90)=0.92, P=0.4688 |  |
|  | **Water intake (ml)**  **Figure 2C** | Treatment: F(1,18)=1.95, P=0.1800 |  |
|  |  | Time: F(5,90)=5.55, P=0.0002 |  |
|  |  | Interaction: F(5,90)=0.59, P=0.7125 |  |
|  | **Total intake (ml)**  **Figure 2D** | Treatment: F(1,18)=4.23, P=0.0546 |  |
|  |  | Time: F(5,90)=4.89, P=0.0005 |  |
|  |  | Interaction: F(5,90)=0.38, P=0.8619 |  |
|  | **Food intake (g)**  **Figure 2E** | Treatment: F(1,18)=0.05, P=0.8309 |  |
|  |  | Time: F(5,90)=8.97, P<0.0001 |  |
|  |  | Interaction: F(5,90)=0.56, P=0.7333 |  |
|  | **Body weight gain (%)**  **Figure 2F** | Treatment: F(1,18)=5.28, P=0.0338 | n.s., s28-33 |
|  |  | Time: F(2,36)=68.92, P<0.0001 |  |
|  |  | Interaction: F(2,36)=0.03, P=0.9684 |  |

Statistical analysis of the effects of nine weeks of dulaglutide treatment (drinking session 1-27) and treatment discontinuation (drinking session 28-33) on all measured parameters in female rats; s: drinking session.

**Supplementary Table 4. Effects of five weeks of dulaglutide treatment on consummatory behaviour in male rats**

|  |  | **Two-way ANOVA, repeated measures** | **Bonferroni post-hoc test** |
| --- | --- | --- | --- |
| **Treatment** | **Alcohol intake (g/kg)**  **Figure 3A** | Treatment: F(1,18)=9.96, P=0.0055 | P<0.05, s1-2, s4-5, s13 |
|  |  | Time: F(14,252)=11.13, P<0.0001 |  |
|  |  | Interaction: F(14,252)=1.02, P=0.4376 |  |
|  | **Alcohol preference (%)**  **Figure 3B** | Treatment: F(1,18)=7.80, P=0.0120 | P<0.05, s4-5 |
|  |  | Time: F(14,252)=3.88, P<0.0001 |  |
|  |  | Interaction: F(14,252)=1.01, P=0.4489 |  |
|  | **Water intake (ml)**  **Figure 3C** | Treatment: F(1,18)=4.51, P=0.0478 | P<0.05, s4-5 |
|  |  | Time: F(14,252)=5.08, P<0.0001 |  |
|  |  | Interaction: F(14,252)=1.45, P=0.1304 |  |
|  | **Total intake (ml)**  **Figure 3D** | Treatment: F(1,18)=0.07, P=0.7964 | n.s., s1-15 |
|  |  | Time: F(14,252)=4.11, P<0.0001 |  |
|  |  | Interaction: F(14,252)=1.90, P=0.0268 |  |
|  | **Food intake (g)**  **Figure 3E** | Treatment: F(1,18)=0.38, P=0.5433 | n.s., s1-15 |
|  |  | Time: F(14,252)=5.87, P<0.0001 |  |
|  |  | Interaction: F(14,252)=3.15, P=0.0003 |  |
|  | **Body weight change (%)**  **Figure 3F** | Treatment: F(1,18)=0.75, P=0.3990 | n.s., s1-15 |
|  |  | Time: F(4,72)=1222.00, P<0.0001 |  |
|  |  | Interaction: F(4,72)=3.45, P=0.0123 |  |
| **Discontinued treatment** | **Alcohol intake (g/kg)**  **Figure 3A** | Treatment: F(1,18)=1.14, P=0.3001 | n.s., s16-33 |
|  |  | Time: F(17,306)=8.57, P<0.0001 |  |
|  |  | Interaction: F(17,306)=3.07, P<0.0001 |  |
|  | **Alcohol preference (%)**  **Figure 3B** | Treatment: F(1,18)=0.02, P=0.8860 | n.s., s16-33 |
|  |  | Time: F(17,306)=12.11, P<0.0001 |  |
|  |  | Interaction: F(17,306)=1.83, P=0.0245 |  |
|  | **Water intake (ml)**  **Figure 3C** | Treatment: F(1,18)=0.00, P=0.9551 |  |
|  |  | Time: F(17,306)=10.46, P<0.0001 |  |
|  |  | Interaction: F(17,306)=1.38, P=0.1449 |  |
|  | **Total intake (ml)**  **Figure 3D** | Treatment: F(1,18)=0.41, P=0.5292 |  |
|  |  | Time: F(17,306)=7.85, P<0.0001 |  |
|  |  | Interaction: F(17,306)=1.27, P=0.2078 |  |
|  | **Food intake (g)**  **Figure 3E** | Treatment: F(1,18)=1.51, P=0.2340 | n.s., s16-33 |
|  |  | Time: F(17,306)=20.57, P<0.0001 |  |
|  |  | Interaction: F(17,306)=1.66, P=0.0492 |  |
|  | **Body weight change (%)**  **Figure 3F** | Treatment: F(1,18)=1.46, P=0.2433 | n.s., s16-33 |
|  |  | Time: F(6,108)=577.00, P<0.0001 |  |
|  |  | Interaction: F(6,108)=3.12, P=0.0074 |  |

Statistical analysis of the effects of five weeks of dulaglutide treatment (drinking session 1-15) and treatment discontinuation (drinking session 16-33) on all measured parameters in male rats; s: drinking session.

**Supplementary Table 5. Effects of five weeks of dulaglutide treatment on consummatory behaviour in female rats**

|  |  | **Two-way ANOVA, repeated measures** | **Bonferroni post-hoc test** |
| --- | --- | --- | --- |
| **Treatment** | **Alcohol intake (g/kg)**  **Figure 4A** | Treatment: F(1,18)=12.52, P=0.0025 | P<0.05, s4-5, s13  P<0.01, s14 |
|  |  | Time: F(14,252)=4.32, P<0.0001 |  |
|  |  | Interaction: F(14,252)=1.39, P=0.1574 |  |
|  | **Alcohol preference (%)**  **Figure 4B** | Treatment: F(1,18)=24.42, P=0.0001 | P<0.01, s1-2, s13-15  P<0.001, s4-5, s10-11 |
|  |  | Time: F(14,252)=8.81, P<0.0001 |  |
|  |  | Interaction: F(14,252)=1.42, P=0.1434 |  |
|  | **Water intake (ml)**  **Figure 4C** | Treatment: F(1,18)=25.07, P<0.0001 | P<0.05, s2, s4-5, s7-9, s15  P<0.01, s6, s13  P<0.001, s10-11, s14 |
|  |  | Time: F(14,252)=1.94, P=0.0230 |  |
|  |  | Interaction: F(14,252)=1.06, P=0.3925 |  |
|  | **Total intake (ml)**  **Figure 4D** | Treatment: F(1,18)=12.43, P=0.0024 | P<0.05, s2 |
|  |  | Time: F(14,252)=1.87, P=0.0299 |  |
|  |  | Interaction: F(14,252)=0.59, P=0.8719 |  |
|  | **Food intake (g)**  **Figure 4E** | Treatment: F(1,18)=0.88, P=0.3604 | n.s., s1-15 |
|  |  | Time: F(14,252)=6.86, P<0.0001 |  |
|  |  | Interaction: F(14,252)=3.30, P<0.0001 |  |
|  | **Body weight change (%)**  **Figure 4F** | Treatment: F(1,18)=2.10, P=0.1646 |  |
|  |  | Time: F(4,72)=116.40, P<0.0001 |  |
|  |  | Interaction: F(4,72)=0.08, P=0.9887 |  |
| **Discontinued treatment** | **Alcohol intake (g/kg)**  **Figure 4A** | Treatment: F(1,18)=0.00, P=0.9796 |  |
|  |  | Time: F(17,306)=4.26, P<0.0001 |  |
|  |  | Interaction: F(17,306)=1.31, P=0.1839 |  |
|  | **Alcohol preference (%)**  **Figure 4B** | Treatment: F(1,18)=0.00, P=0.9489 |  |
|  |  | Time: F(17,306)=8.96, P<0.0001 |  |
|  |  | Interaction: F(17,306)=0.99, P=0.4719 |  |
|  | **Water intake (ml)**  **Figure 4C** | Treatment: F(1,18)=0.16, P=0.6934 |  |
|  |  | Time: F(17,306)=6.49, P<0.0001 |  |
|  |  | Interaction: F(17,306)=1.46, P=0.1095 |  |
|  | **Total intake (ml)**  **Figure 4D** | Treatment: F(1,18)=0.07, P=0.7914 | n.s., s16-33 |
|  |  | Time: F(17,306)=6.91, P<0.0001 |  |
|  |  | Interaction: F(17,306)=1.81, P=0.0264 |  |
|  | **Food intake (g)**  **Figure 4E** | Treatment: F(1,18)=3.43, P=0.0805 |  |
|  |  | Time: F(17,306)=5.94, P<0.0001 |  |
|  |  | Interaction: F(17,306)=0.65, P=0.8506 |  |
|  | **Body weight change (%)**  **Figure 4F** | Treatment: F(1,18)=3.36, P=0.0836 | n.s., s16-33 |
|  |  | Time: F(6,108)=92.60, P<0.0001 |  |
|  |  | Interaction: F(6,108)=3.65, P=0.0025 |  |

Statistical analysis of the effects of five weeks of dulaglutide treatment (drinking session 1-15) and treatment discontinuation (drinking session 16-33) on all measured parameters in female rats; s: drinking session.

**Supplementary Table 6. Differences in the effects of dulaglutide on alcohol intake between male and female rats.**

|  |  | Mean | SEM | P-value (unpaired t-test) |
| --- | --- | --- | --- | --- |
| Nine weeks of treatment | Males, n=25 | 3.84 | 0.09 | 0.0001 |
|  | Females, n=10 | 1.96 | 0.21 |  |
| Five weeks of treatment | Males, n=10 | 2.18 | 0.13 | 0.0007 |
|  | Females, n=10 | 1.43 | 0.13 |  |

Data are presented as mean ±SEM, calculated as the difference in average of alcohol intake over time between vehicle and dulaglutide treatment.

**Supplementary Table 7. Effects of discontinuation of dulaglutide treatment on the *ex vivo* levels of monoamines and their metabolites in brain areas of male rats consuming ethanol.**

|  |  | **DOPAC** | **Dopamine** | **DOPAC/**  **dopamine** | **5-HIAA** | **Serotonin** | **5-HIAA/**  **serotonin** | **Noradrenaline** | **3-MT** |
| --- | --- | --- | --- | --- | --- | --- | --- | --- | --- |
| **VTA** | Veh | 2.1±0.8 | 4.0±0.8 | 0.6±0.2 | 1.3±0.3 | 1.8±0.2 | 0.9±0.2 | 3.3±0.4 | 0.07±0.02 |
|  | Dul | 1.5±0.7 | 4.2±0.8 | 0.3±0.1 | 1.1±0.2 | 1.9±0.2 | 0.6±0.1 | 2.7±0.2 | 0.09±0.02 |
|  | *p-value* | 0.5802 | 0.8588 | 0.3053 | 0.4518 | 0.5297 | 0.2607 | 0.1865 | 0.5805 |
| **PFC** | Veh | 0.06±0.02 | 0.1±0.0 | 0.4±0.1 | 1.4±0.2 | 2.7±0.3 | 0.6±0.1 | 1.2±0.05 | n.d. |
|  | Dul | 0.08±0.01 | 0.2±0.0 | 0.5±0.1 | 1.2±0.1 | 2.7±0.2 | 0.4±0.1 | 1.2±0.1 | n.d. |
|  | *p-value* | 0.5273 | 0.1912 | 0.9537 | 0.2160 | 0.9915 | 0.2437 | 0.3930 |  |
| **HIP** | Veh | n.d. | 0.02±0.01 | n.d. | 1.3±0.1 | 1.6±0.1 | 0.9±0.1 | 2.1±0.1 | n.d. |
|  | Dul | n.d. | 0.02±0.01 | n.d. | 1.5±0.1 | 1.5±0.1 | 1.0±0.1 | 2.4±0.1 | n.d. |
|  | *p-value* |  | 0.5222 |  | 0.3641 | 0.6201 | 0.2461 | 0.0834 |  |
| **NAc** | Veh | 8.9±1.7 | 20.8±2.1 | 0.6±0.2 | 2.9±0.4 | 4.0±0.4 | 0.9±0.2 | 4.1±0.8 | 0.6±0.1 |
|  | Dul | 7.4±1.6 | 24.3±1.8 | 0.3±0.1 | 2.7±0.4 | 5.0±0.3 | 0.6±0.1 | 4.6±0.9 | 0.7±0.1 |
|  | *p-value* | 0.5234 | 0.2165 | 0.3166 | 0.6719 | 0.0534 | 0.1777 | 0.6774 | 0.3089 |
| **STRI** | Veh | 10.4±1.7 | 48.5±4.7 | 0.3±0.1 | 1.7±0.2 | 1.7±0.2 | 1.2±0.2 | 0.13±0.04 | 1.5±0.1 |
|  | Dul | 11.6±2.9 | 46.4±4.7 | 0.3±0.1 | 2.0±0.1 | 1.5±0.2 | 1.5±0.2 | 0.10±0.03 | 1.4±0.1 |
|  | *p-value* | 0.7144 | 0.7529 | 0.5063 | 0.1444 | 0.5289 | 0.3300 | 0.5467 | 0.9520 |
| **AMY** | Veh | 2.1±0.8 | 4.0±0.8 | 0.6±0.2 | 1.3±0.3 | 1.7±0.2 | 0.9±0.2 | 3.3±0.4 | 0.07±0.03 |
|  | Dul | 1.5±0.7 | 4.2±0.7 | 0.3±0.1 | 1.1±0.2 | 1.9±0.2 | 0.6±0.1 | 2.7±0.2 | 0.09±0.02 |
|  | *p-value* | 0.5802 | 0.8588 | 0.3053 | 0.4518 | 0.5297 | 0.2607 | 0.1865 | 0.5805 |

Dulaglutide (Dul) did not affect the levels of the measured neurotransmitters compared to vehicle (Veh), in the investigated areas: Ventral tegmental area (VTA), prefrontal cortex (PFC), hippocampus (HIP), nucleus accumbens (NAc), striatum (STRI), and amygdala (AMY)**.** Data are presented as mean±SEM, n.d.: not detected. **Supplementary Table 8. Effects of discontinuation of dulaglutide treatment on the *ex vivo* levels of monoamines and their metabolites in brain areas of female rats consuming ethanol.**

|  |  | **DOPAC** | **Dopamine** | **DOPAC/**  **dopamine** | **5-HIAA** | **Serotonin** | **5-HIAA/**  **serotonin** | **Noradrenaline** | **3-MT** |
| --- | --- | --- | --- | --- | --- | --- | --- | --- | --- |
| **VTA** | Veh | 0.17±0.08 | 0.6±0.2 | 0.3±0.1 | 2.2±0.2 | 2.5±0.2 | 0.9±0.1 | 1.9±0.3 | n.d. |
|  | Dul | 0.08±0.03 | 0.8±0.2 | 0.2±0.1 | 1.9±0.2 | 2.1±0.3 | 1.0±0.1 | 1.7±0.3 | n.d. |
|  | *p-value* | 0.2637 | 0.5503 | 0.2181 | 0.2932 | 0.2501 | 0.5604 | 0.7007 |  |
| **PFC** | Veh | 0.07±0.02 | 0.3±0.0 | 0.3±0.1 | 1.1±0.1 | 3.7±0.2 | 0.3±0.0 | 1.9±0.2 | n.d. |
|  | Dul | 0.08±0.03 | 0.3±0.0 | 0.3±0.1 | 0.9±0.1 | 3.4±0.3 | 0.3±0.0 | 1.8±0.2 | n.d. |
|  | *p-value* | 0.8447 | 0.3023 | 0.7348 | 0.2700 | 0.4636 | 0.8645 | 0.6499 |  |
| **HIP** | Veh | n.d. | 0.02±0.01 | n.d. | 1.2±0.0 | 1.5±0.1 | 0.8±0.0 | 2.0±0.1 | n.d. |
|  | Dul | n.d. | 0.02±0.00 | n.d. | 1.2±0.1 | 1.4±0.1 | 0.8±0.1 | 1.9±0.2 | n.d. |
|  | *p-value* |  | 0.8856 |  | 0.7562 | 0.7397 | 0.8958 | 0.6944 |  |
| **NAc** | Veh | 3.6±0.3 | 24.7±1.6 | 0.14±0.00 | 2.1±0.1 | 5.8±0.8 | 0.39±0.02 | 2.7±0.5 | 0.6±0.1 |
|  | Dul | 3.7±0.2 | 24.6±1.2 | 0.15±0.01 | 2.3±0.1 | 5.2±0.3 | 0.43±0.02 | 4.2±0.9 | 0.7±0.1 |
|  | *p-value* | 0.6354 | 0.9872 | 0.3780 | 0.2269 | 0.1529 | 0.0908 | 0.1643 | 0.2819 |
| **STRI** | Veh | 4.9±0.7 | 54.6±4.1 | 0.09±0.01 | 1.8±0.1 | 2.1±0.1 | 0.9±0.1 | 0.06±0.02 | 1.6±0.1 |
|  | Dul | 4.1±0.4 | 50.9±2.8 | 0.08±0.00 | 1.6±0.1 | 2.0±0.1 | 0.8±0.0 | 0.1±0.1 | 1.7±0.2 |
|  | *p-value* | 0.3391 | 0.4586 | 0.4135 | 0.2854 | 0.6861 | 0.2803 | 0.3784 | 0.5131 |
| **AMY** | Veh | 0.1±0.0 | 1.6±0.2 | 0.13±0.06 | 1.0±0.2 | 2.1±0.3 | 0.5±0.1 | 2.1±0.2 | n.d. |
|  | Dul | 0.1±0.1 | 1.2±0.7 | 0.8±0.04 | 0.7±0.1 | 1.5±0.3 | 0.6±0.1 | 1.6±0.2 | n.d. |
|  | *p-value* | 0.9399 | 0.6678 | 0.3841 | 0.1617 | 0.2496 | 0.7674 | 0.0598 |  |

Dulaglutide (Dul) did not affect the levels of the measured neurotransmitters compared to vehicle (Veh), in the investigated areas: Ventral tegmental area (VTA), prefrontal cortex (PFC), hippocampus (HIP), nucleus accumbens (NAc), striatum (STRI), and amygdala (AMY)**.** Data are presented as mean±SEM, n.d.: not detected.
