# Supplementary material for: Long-term treatment with a glucagon-like peptide-1 receptor agonist reduces ethanol intake in male and female rats
Source: Transl Psychiatry. 2020 Jul 16;10:238. doi: 10.1038/s41398-020-00923-1 (PMC7367312; doi:10.1038/s41398-020-00923-1)
Supplement: Supplementary file 2 — Supplementary Figures [file 41398_2020_923_MOESM2_ESM.pptx]

## Slide 1
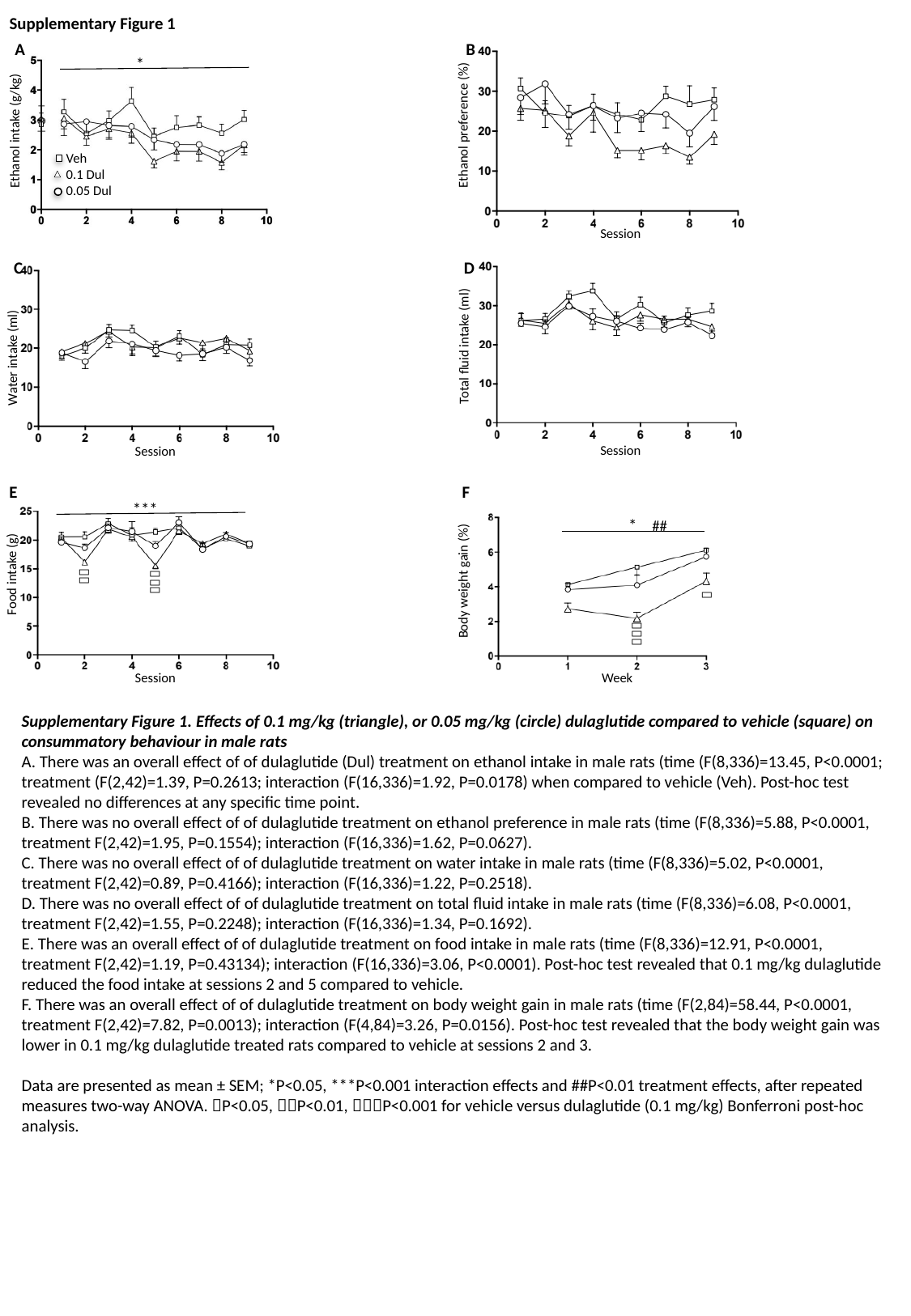

Supplementary Figure 1
A B
*
Ethanol preference (%)
Ethanol intake (g/kg)
Veh
0.1 Dul
0.05 Dul
Session
C D
Total fluid intake (ml)
Water intake (ml)
Session
Session
E F
***
* ##
Food intake (g)

Body weight gain (%)



Session
Week
Supplementary Figure 1. Effects of 0.1 mg/kg (triangle), or 0.05 mg/kg (circle) dulaglutide compared to vehicle (square) on consummatory behaviour in male rats
A. There was an overall effect of of dulaglutide (Dul) treatment on ethanol intake in male rats (time (F(8,336)=13.45, P<0.0001; treatment (F(2,42)=1.39, P=0.2613; interaction (F(16,336)=1.92, P=0.0178) when compared to vehicle (Veh). Post-hoc test revealed no differences at any specific time point.
B. There was no overall effect of of dulaglutide treatment on ethanol preference in male rats (time (F(8,336)=5.88, P<0.0001, treatment F(2,42)=1.95, P=0.1554); interaction (F(16,336)=1.62, P=0.0627).
C. There was no overall effect of of dulaglutide treatment on water intake in male rats (time (F(8,336)=5.02, P<0.0001, treatment F(2,42)=0.89, P=0.4166); interaction (F(16,336)=1.22, P=0.2518).
D. There was no overall effect of of dulaglutide treatment on total fluid intake in male rats (time (F(8,336)=6.08, P<0.0001, treatment F(2,42)=1.55, P=0.2248); interaction (F(16,336)=1.34, P=0.1692).
E. There was an overall effect of of dulaglutide treatment on food intake in male rats (time (F(8,336)=12.91, P<0.0001, treatment F(2,42)=1.19, P=0.43134); interaction (F(16,336)=3.06, P<0.0001). Post-hoc test revealed that 0.1 mg/kg dulaglutide reduced the food intake at sessions 2 and 5 compared to vehicle.
F. There was an overall effect of of dulaglutide treatment on body weight gain in male rats (time (F(2,84)=58.44, P<0.0001, treatment F(2,42)=7.82, P=0.0013); interaction (F(4,84)=3.26, P=0.0156). Post-hoc test revealed that the body weight gain was lower in 0.1 mg/kg dulaglutide treated rats compared to vehicle at sessions 2 and 3.
Data are presented as mean ± SEM; *P<0.05, ***P<0.001 interaction effects and ##P<0.01 treatment effects, after repeated measures two-way ANOVA. P<0.05, P<0.01, P<0.001 for vehicle versus dulaglutide (0.1 mg/kg) Bonferroni post-hoc analysis.

## Slide 2
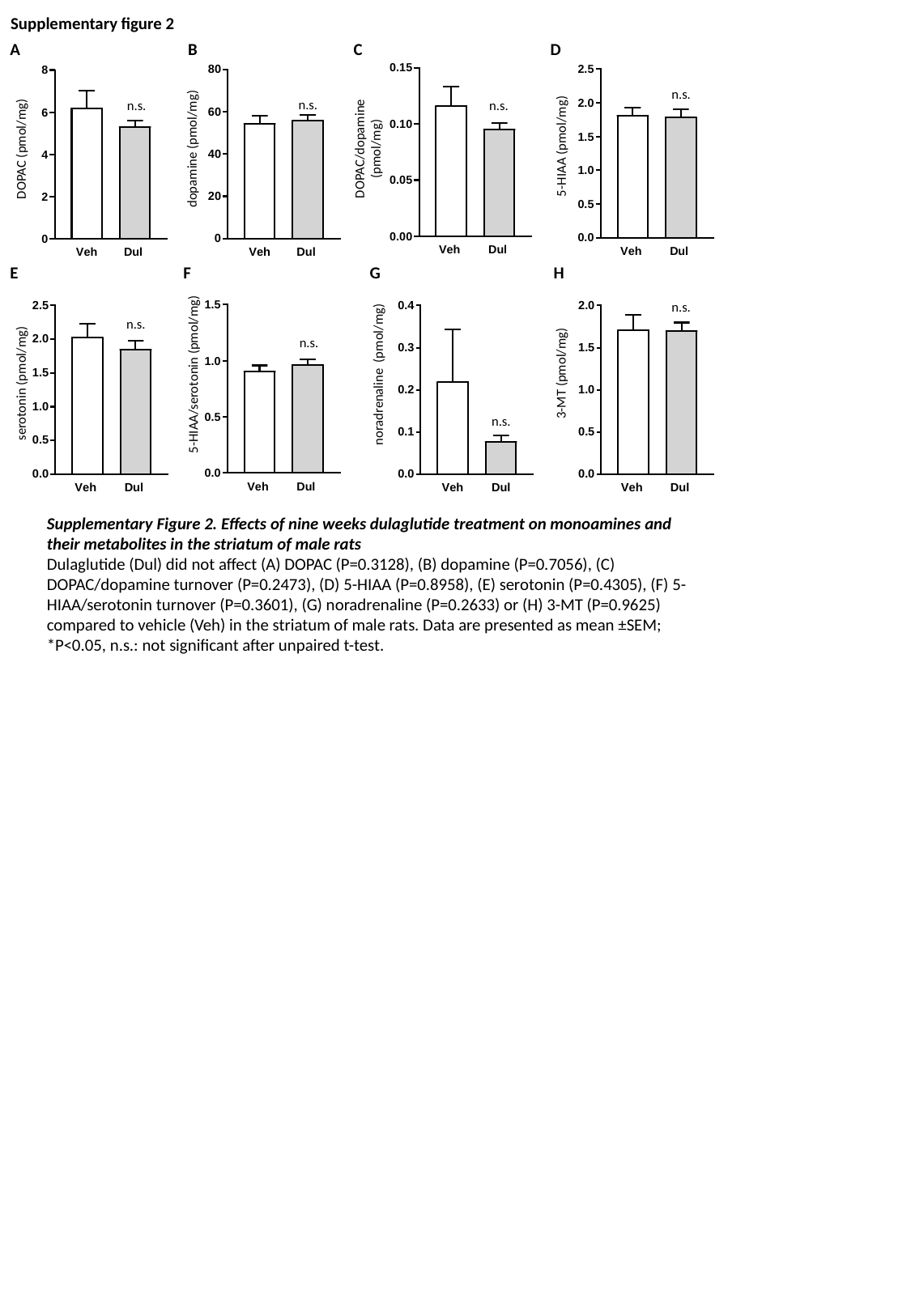

Supplementary figure 2
A
B
C
D
n.s.
n.s.
n.s.
n.s.
DOPAC/dopamine
(pmol/mg)
5-HIAA (pmol/mg)
DOPAC (pmol/mg)
dopamine (pmol/mg)
E
F
G
H
n.s.
n.s.
n.s.
3-MT (pmol/mg)
5-HIAA/serotonin (pmol/mg)
noradrenaline (pmol/mg)
serotonin (pmol/mg)
n.s.
Supplementary Figure 2. Effects of nine weeks dulaglutide treatment on monoamines and their metabolites in the striatum of male rats
Dulaglutide (Dul) did not affect (A) DOPAC (P=0.3128), (B) dopamine (P=0.7056), (C) DOPAC/dopamine turnover (P=0.2473), (D) 5-HIAA (P=0.8958), (E) serotonin (P=0.4305), (F) 5-HIAA/serotonin turnover (P=0.3601), (G) noradrenaline (P=0.2633) or (H) 3-MT (P=0.9625) compared to vehicle (Veh) in the striatum of male rats. Data are presented as mean ±SEM; *P<0.05, n.s.: not significant after unpaired t-test.

## Slide 3
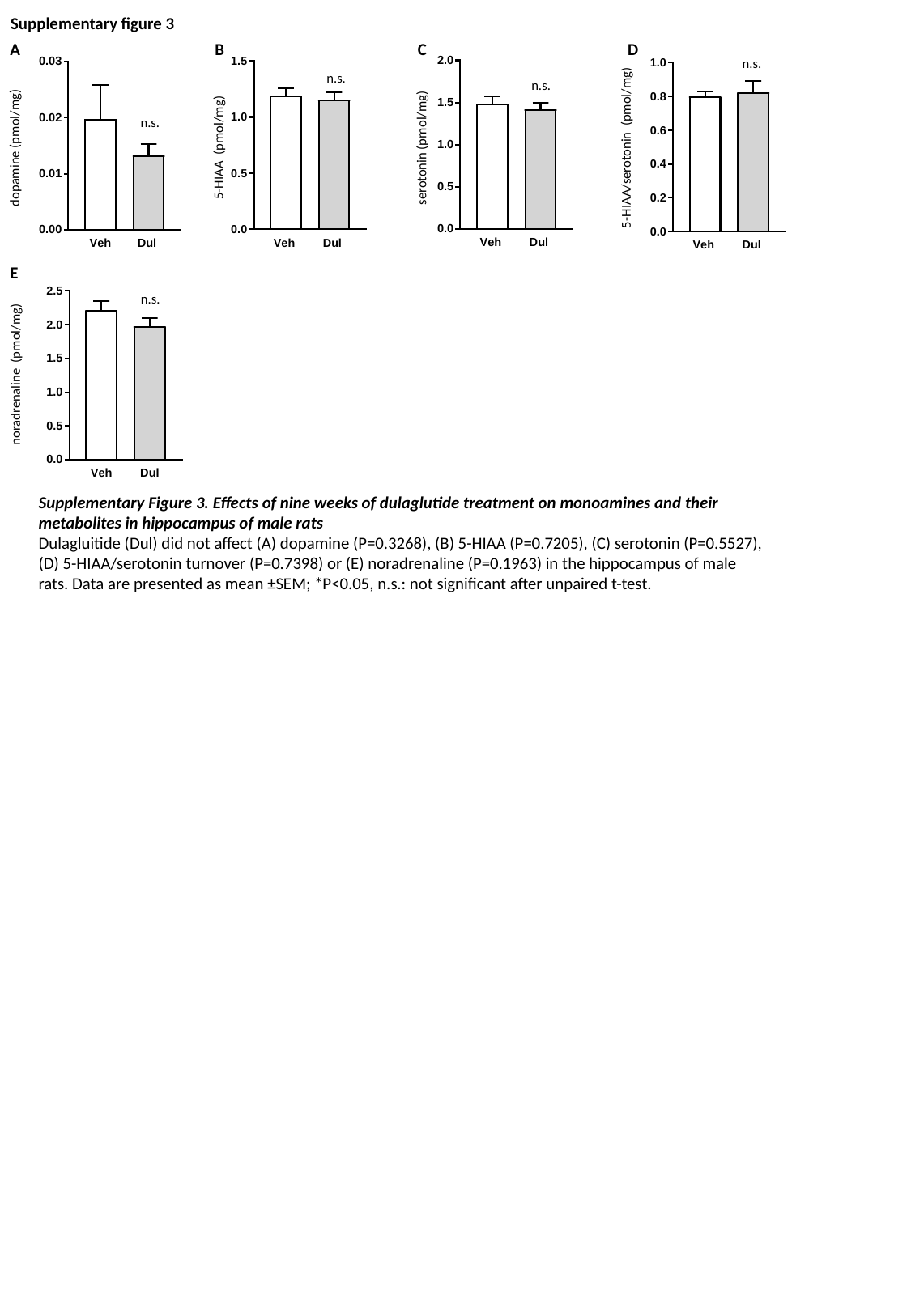

Supplementary figure 3
A
B
C
D
n.s.
n.s.
n.s.
n.s.
dopamine (pmol/mg)
5-HIAA (pmol/mg)
serotonin (pmol/mg)
5-HIAA/serotonin (pmol/mg)
E
n.s.
noradrenaline (pmol/mg)
Supplementary Figure 3. Effects of nine weeks of dulaglutide treatment on monoamines and their metabolites in hippocampus of male rats
Dulagluitide (Dul) did not affect (A) dopamine (P=0.3268), (B) 5-HIAA (P=0.7205), (C) serotonin (P=0.5527), (D) 5-HIAA/serotonin turnover (P=0.7398) or (E) noradrenaline (P=0.1963) in the hippocampus of male rats. Data are presented as mean ±SEM; *P<0.05, n.s.: not significant after unpaired t-test.

## Slide 4
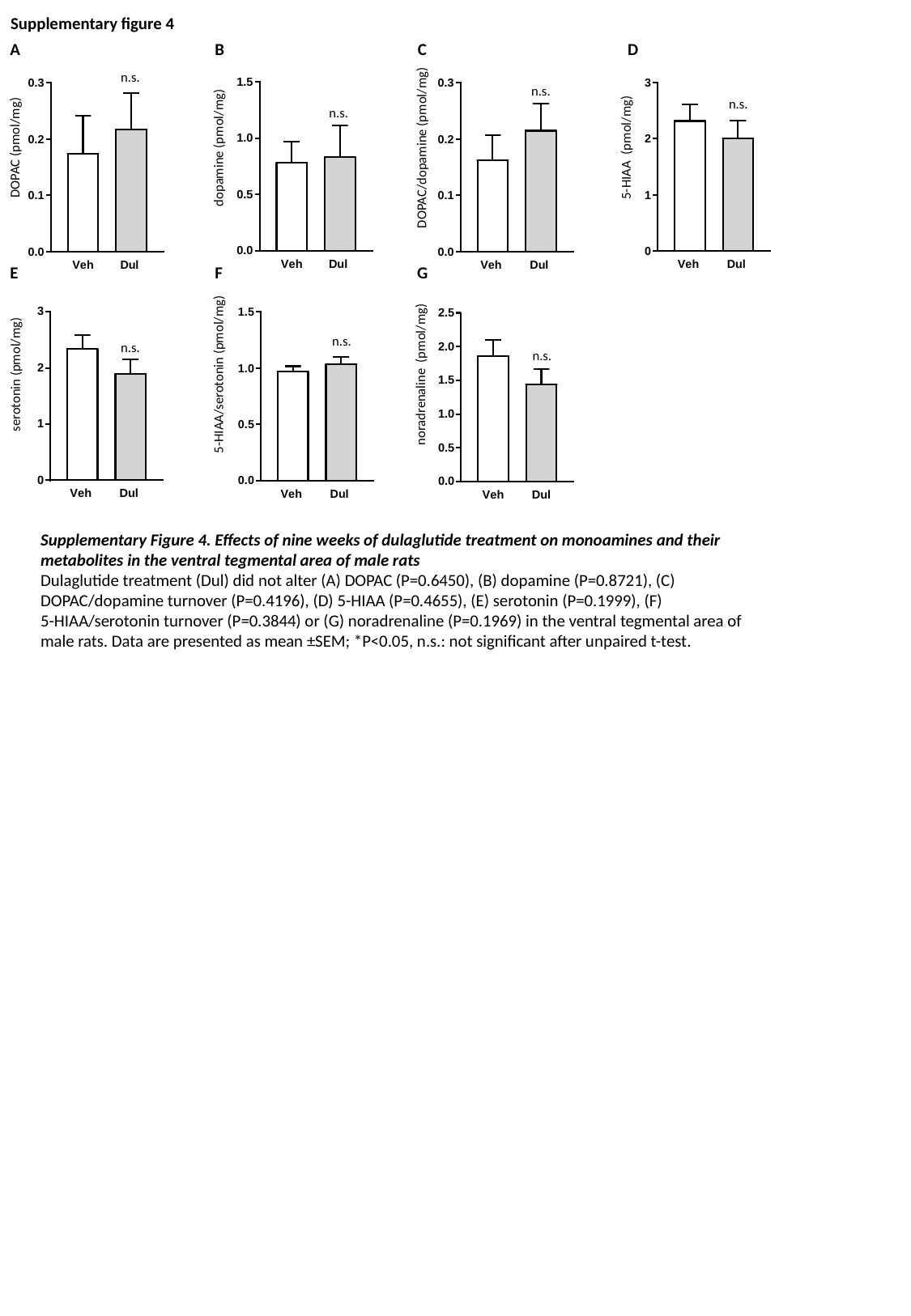

Supplementary figure 4
A
B
C
D
n.s.
n.s.
n.s.
n.s.
DOPAC (pmol/mg)
dopamine (pmol/mg)
DOPAC/dopamine (pmol/mg)
5-HIAA (pmol/mg)
E
F
G
n.s.
n.s.
n.s.
serotonin (pmol/mg)
5-HIAA/serotonin (pmol/mg)
noradrenaline (pmol/mg)
Supplementary Figure 4. Effects of nine weeks of dulaglutide treatment on monoamines and their metabolites in the ventral tegmental area of male rats
Dulaglutide treatment (Dul) did not alter (A) DOPAC (P=0.6450), (B) dopamine (P=0.8721), (C) DOPAC/dopamine turnover (P=0.4196), (D) 5-HIAA (P=0.4655), (E) serotonin (P=0.1999), (F) 5-HIAA/serotonin turnover (P=0.3844) or (G) noradrenaline (P=0.1969) in the ventral tegmental area of male rats. Data are presented as mean ±SEM; *P<0.05, n.s.: not significant after unpaired t-test.

## Slide 5
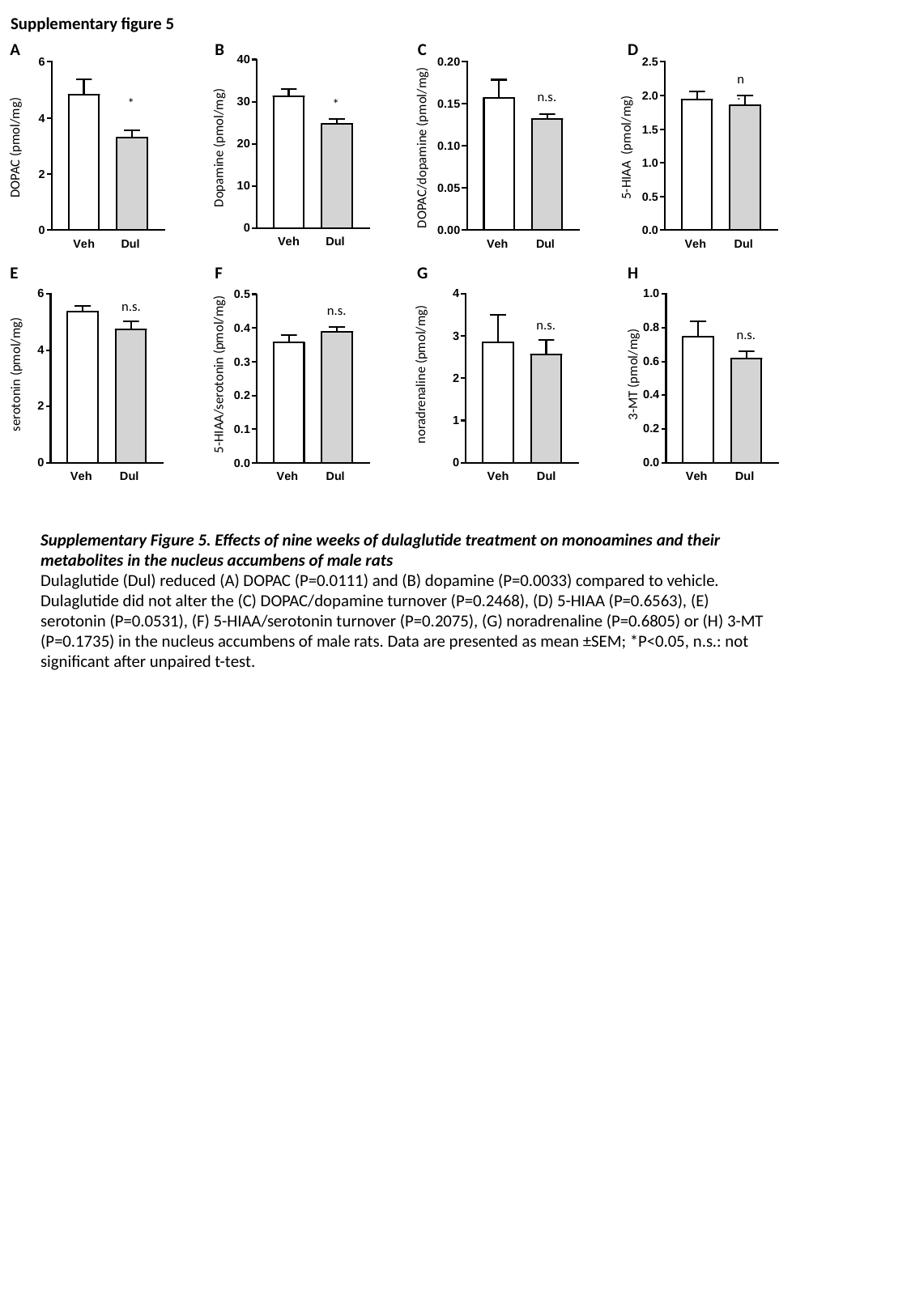

Supplementary figure 5
A
B
C
D
n.s.
n.s.
*
*
DOPAC (pmol/mg)
Dopamine (pmol/mg)
DOPAC/dopamine (pmol/mg)
5-HIAA (pmol/mg)
E
F
G
H
n.s.
n.s.
n.s.
n.s.
serotonin (pmol/mg)
5-HIAA/serotonin (pmol/mg)
noradrenaline (pmol/mg)
3-MT (pmol/mg)
Supplementary Figure 5. Effects of nine weeks of dulaglutide treatment on monoamines and their metabolites in the nucleus accumbens of male rats
Dulaglutide (Dul) reduced (A) DOPAC (P=0.0111) and (B) dopamine (P=0.0033) compared to vehicle. Dulaglutide did not alter the (C) DOPAC/dopamine turnover (P=0.2468), (D) 5-HIAA (P=0.6563), (E) serotonin (P=0.0531), (F) 5-HIAA/serotonin turnover (P=0.2075), (G) noradrenaline (P=0.6805) or (H) 3-MT (P=0.1735) in the nucleus accumbens of male rats. Data are presented as mean ±SEM; *P<0.05, n.s.: not significant after unpaired t-test.

## Slide 6
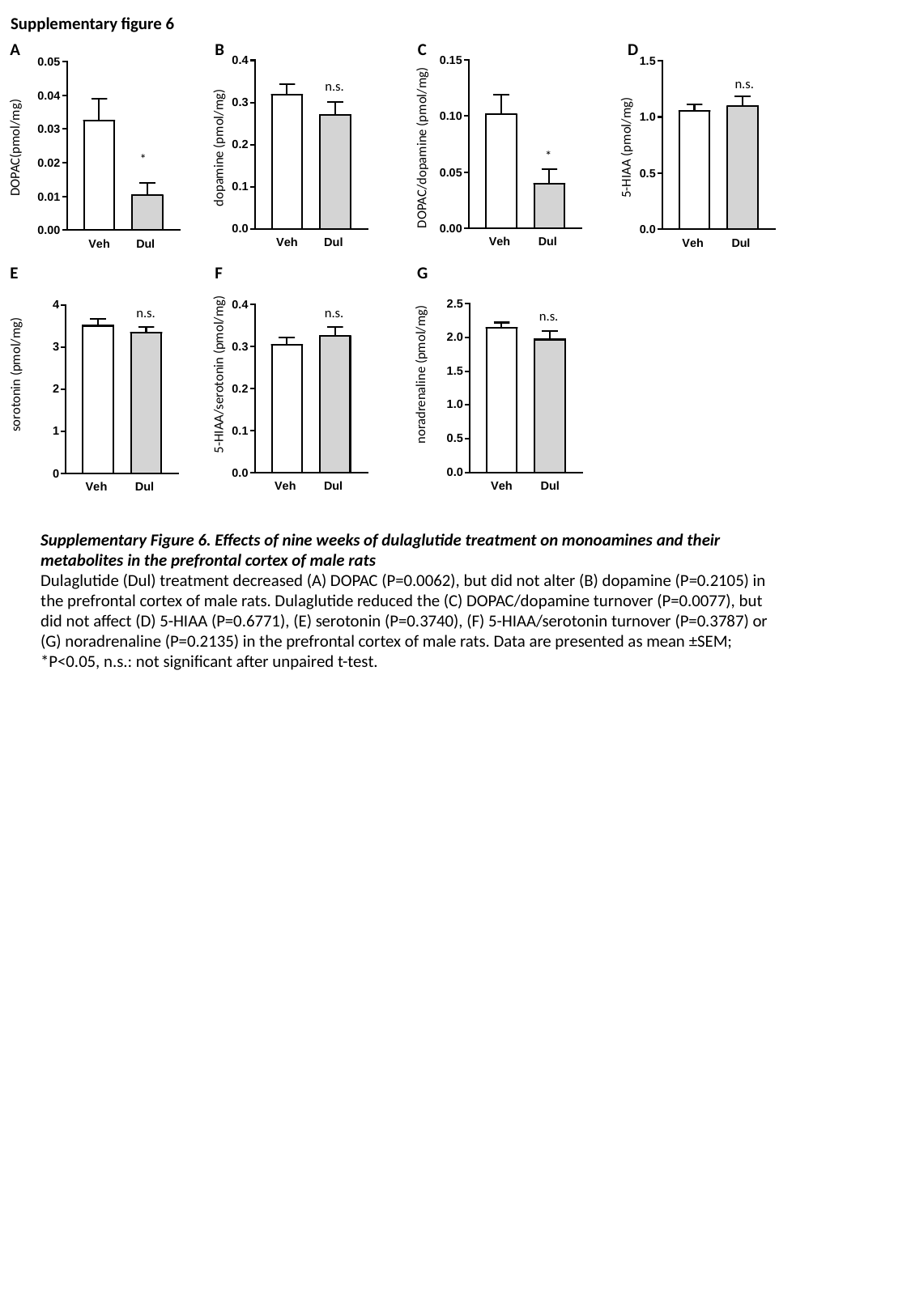

Supplementary figure 6
A
B
C
D
n.s.
n.s.
DOPAC(pmol/mg)
dopamine (pmol/mg)
DOPAC/dopamine (pmol/mg)
5-HIAA (pmol/mg)
*
*
E
F
G
n.s.
n.s.
n.s.
sorotonin (pmol/mg)
5-HIAA/serotonin (pmol/mg)
noradrenaline (pmol/mg)
Supplementary Figure 6. Effects of nine weeks of dulaglutide treatment on monoamines and their metabolites in the prefrontal cortex of male rats
Dulaglutide (Dul) treatment decreased (A) DOPAC (P=0.0062), but did not alter (B) dopamine (P=0.2105) in the prefrontal cortex of male rats. Dulaglutide reduced the (C) DOPAC/dopamine turnover (P=0.0077), but did not affect (D) 5-HIAA (P=0.6771), (E) serotonin (P=0.3740), (F) 5-HIAA/serotonin turnover (P=0.3787) or (G) noradrenaline (P=0.2135) in the prefrontal cortex of male rats. Data are presented as mean ±SEM; *P<0.05, n.s.: not significant after unpaired t-test.

## Slide 7
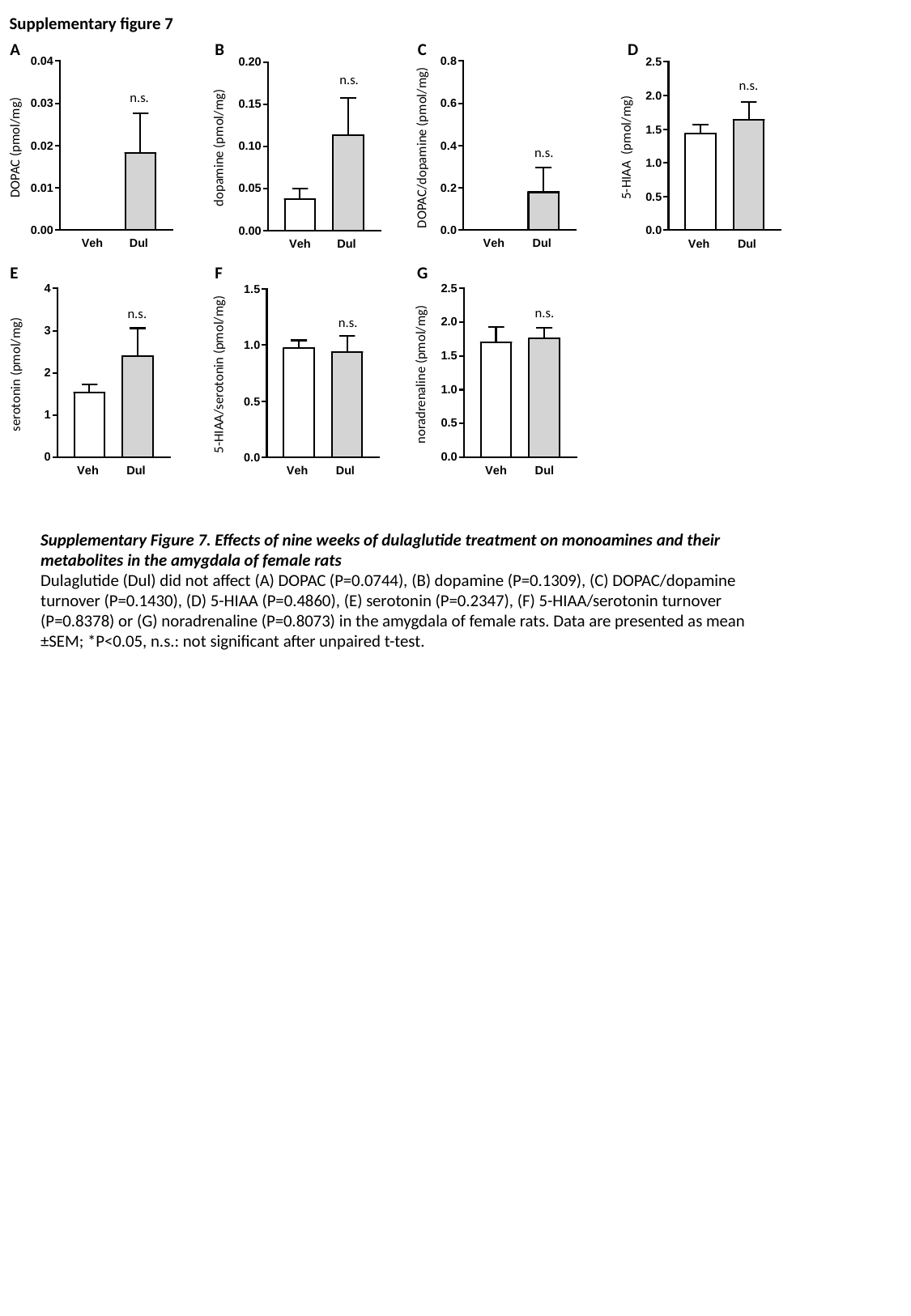

Supplementary figure 7
A
B
C
D
n.s.
n.s.
n.s.
DOPAC (pmol/mg)
dopamine (pmol/mg)
DOPAC/dopamine (pmol/mg)
5-HIAA (pmol/mg)
n.s.
E
F
G
n.s.
n.s.
n.s.
serotonin (pmol/mg)
5-HIAA/serotonin (pmol/mg)
noradrenaline (pmol/mg)
Supplementary Figure 7. Effects of nine weeks of dulaglutide treatment on monoamines and their metabolites in the amygdala of female rats
Dulaglutide (Dul) did not affect (A) DOPAC (P=0.0744), (B) dopamine (P=0.1309), (C) DOPAC/dopamine turnover (P=0.1430), (D) 5-HIAA (P=0.4860), (E) serotonin (P=0.2347), (F) 5-HIAA/serotonin turnover (P=0.8378) or (G) noradrenaline (P=0.8073) in the amygdala of female rats. Data are presented as mean ±SEM; *P<0.05, n.s.: not significant after unpaired t-test.

## Slide 8
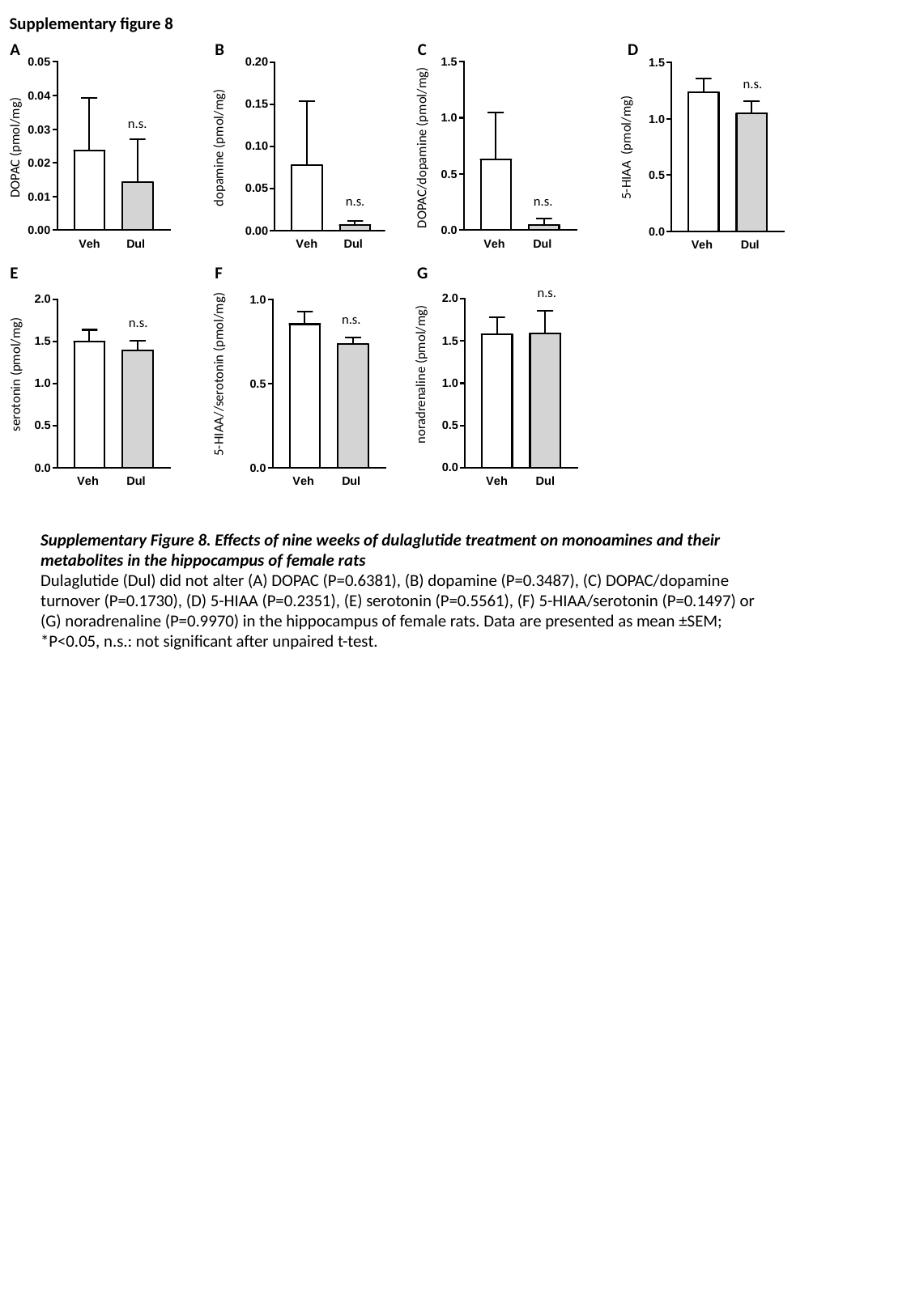

Supplementary figure 8
A
B
C
D
n.s.
n.s.
DOPAC (pmol/mg)
dopamine (pmol/mg)
DOPAC/dopamine (pmol/mg)
5-HIAA (pmol/mg)
n.s.
n.s.
E
F
G
n.s.
n.s.
n.s.
serotonin (pmol/mg)
5-HIAA//serotonin (pmol/mg)
noradrenaline (pmol/mg)
Supplementary Figure 8. Effects of nine weeks of dulaglutide treatment on monoamines and their metabolites in the hippocampus of female rats
Dulaglutide (Dul) did not alter (A) DOPAC (P=0.6381), (B) dopamine (P=0.3487), (C) DOPAC/dopamine turnover (P=0.1730), (D) 5-HIAA (P=0.2351), (E) serotonin (P=0.5561), (F) 5-HIAA/serotonin (P=0.1497) or (G) noradrenaline (P=0.9970) in the hippocampus of female rats. Data are presented as mean ±SEM; *P<0.05, n.s.: not significant after unpaired t-test.

## Slide 9
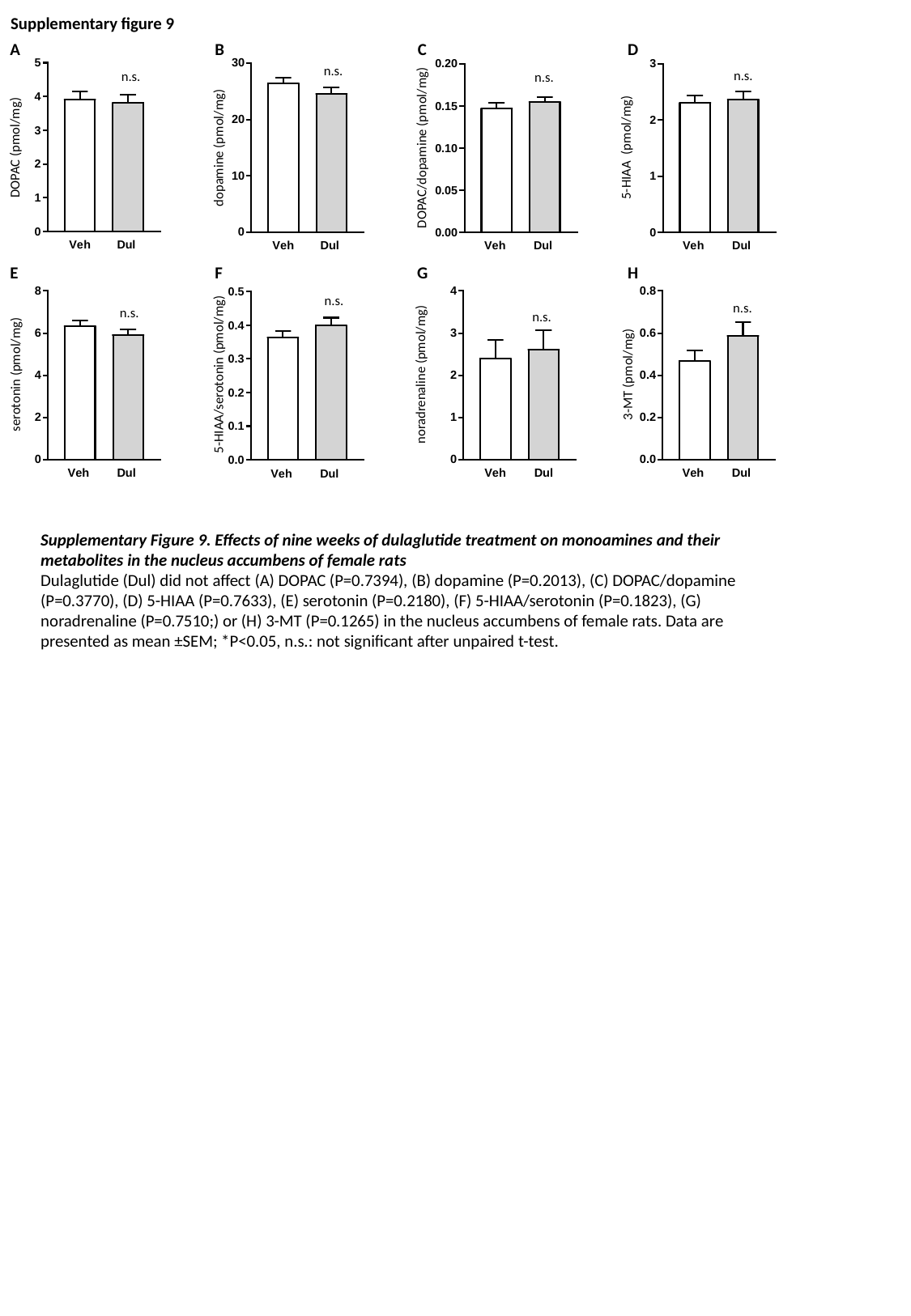

Supplementary figure 9
A
B
C
D
n.s.
n.s.
n.s.
n.s.
n.s.
DOPAC (pmol/mg)
dopamine (pmol/mg)
DOPAC/dopamine (pmol/mg)
5-HIAA (pmol/mg)
E
F
G
H
n.s.
n.s.
n.s.
n.s.
serotonin (pmol/mg)
5-HIAA/serotonin (pmol/mg)
noradrenaline (pmol/mg)
3-MT (pmol/mg)
Supplementary Figure 9. Effects of nine weeks of dulaglutide treatment on monoamines and their metabolites in the nucleus accumbens of female rats
Dulaglutide (Dul) did not affect (A) DOPAC (P=0.7394), (B) dopamine (P=0.2013), (C) DOPAC/dopamine (P=0.3770), (D) 5-HIAA (P=0.7633), (E) serotonin (P=0.2180), (F) 5-HIAA/serotonin (P=0.1823), (G) noradrenaline (P=0.7510;) or (H) 3-MT (P=0.1265) in the nucleus accumbens of female rats. Data are presented as mean ±SEM; *P<0.05, n.s.: not significant after unpaired t-test.

## Slide 10
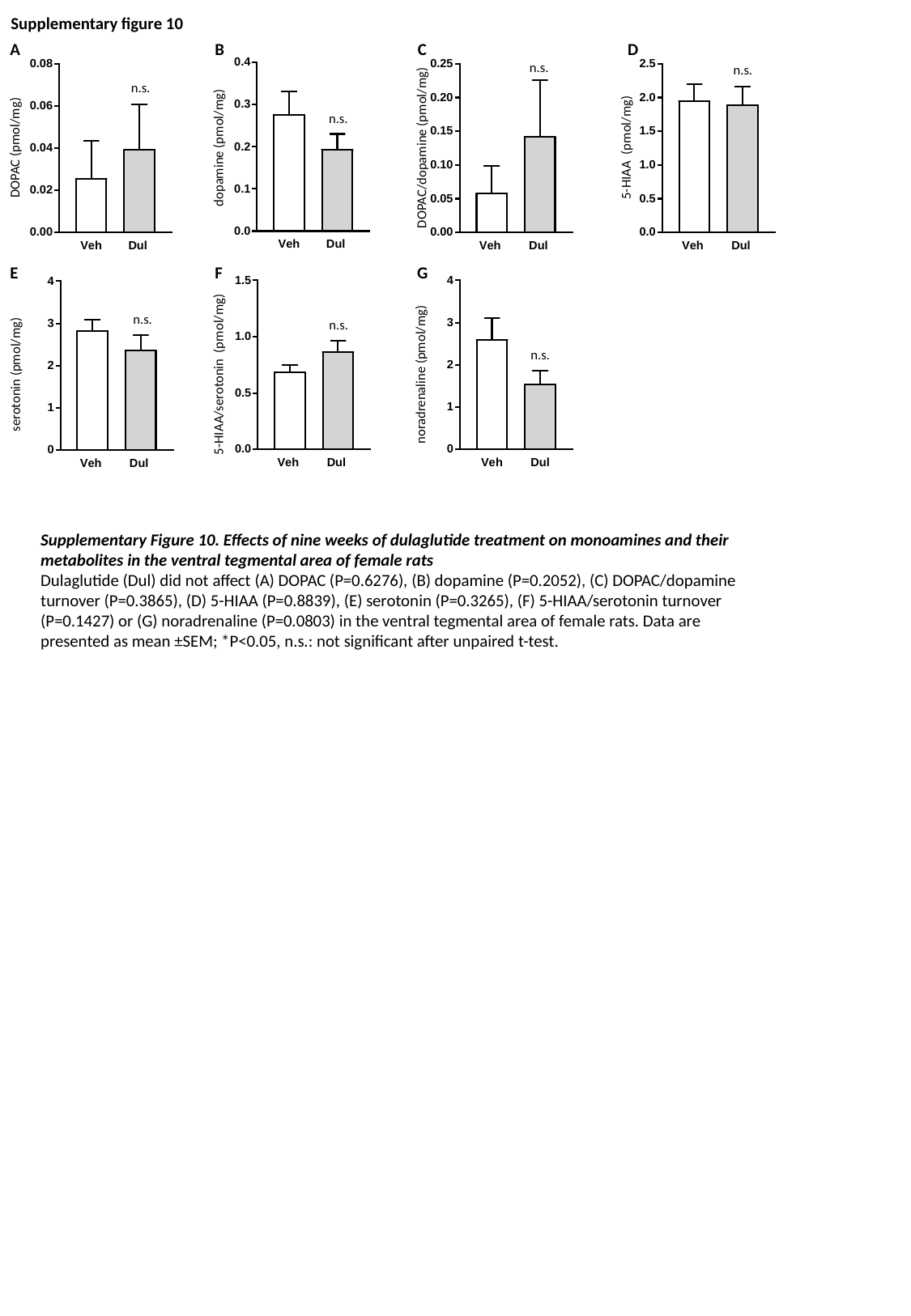

Supplementary figure 10
A
B
C
D
n.s.
n.s.
n.s.
n.s.
DOPAC (pmol/mg)
dopamine (pmol/mg)
DOPAC/dopamine (pmol/mg)
5-HIAA (pmol/mg)
E
F
G
n.s.
n.s.
n.s.
serotonin (pmol/mg)
5-HIAA/serotonin (pmol/mg)
noradrenaline (pmol/mg)
Supplementary Figure 10. Effects of nine weeks of dulaglutide treatment on monoamines and their metabolites in the ventral tegmental area of female rats
Dulaglutide (Dul) did not affect (A) DOPAC (P=0.6276), (B) dopamine (P=0.2052), (C) DOPAC/dopamine turnover (P=0.3865), (D) 5-HIAA (P=0.8839), (E) serotonin (P=0.3265), (F) 5-HIAA/serotonin turnover (P=0.1427) or (G) noradrenaline (P=0.0803) in the ventral tegmental area of female rats. Data are presented as mean ±SEM; *P<0.05, n.s.: not significant after unpaired t-test.

## Slide 11
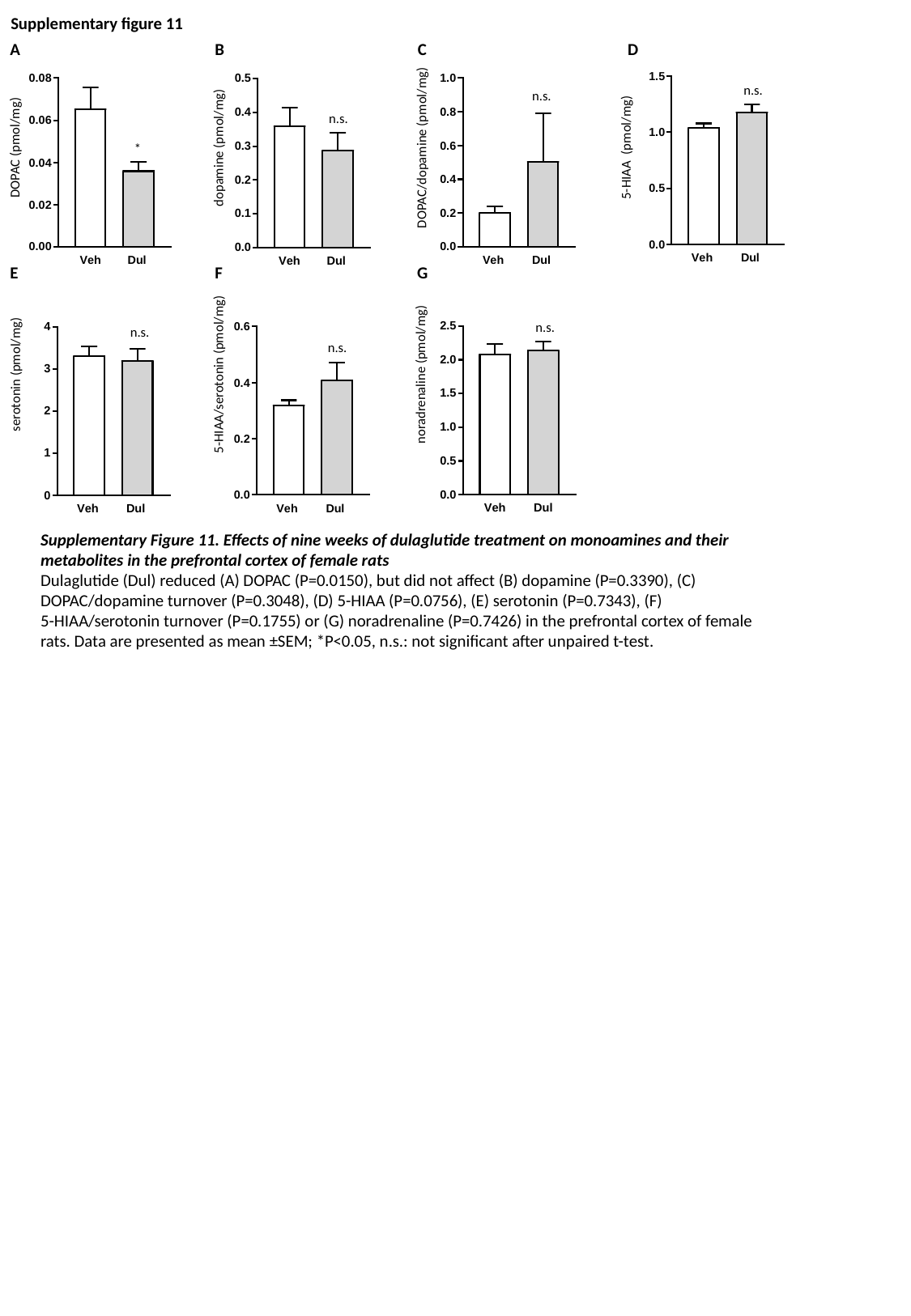

Supplementary figure 11
A
B
C
D
n.s.
n.s.
n.s.
DOPAC (pmol/mg)
dopamine (pmol/mg)
DOPAC/dopamine (pmol/mg)
5-HIAA (pmol/mg)
*
E
F
G
n.s.
n.s.
n.s.
serotonin (pmol/mg)
5-HIAA/serotonin (pmol/mg)
noradrenaline (pmol/mg)
Supplementary Figure 11. Effects of nine weeks of dulaglutide treatment on monoamines and their metabolites in the prefrontal cortex of female rats
Dulaglutide (Dul) reduced (A) DOPAC (P=0.0150), but did not affect (B) dopamine (P=0.3390), (C) DOPAC/dopamine turnover (P=0.3048), (D) 5-HIAA (P=0.0756), (E) serotonin (P=0.7343), (F) 5-HIAA/serotonin turnover (P=0.1755) or (G) noradrenaline (P=0.7426) in the prefrontal cortex of female rats. Data are presented as mean ±SEM; *P<0.05, n.s.: not significant after unpaired t-test.
